# Supplementary material for: “It’s the poverty”—Stakeholder perspectives on barriers to secondary education in rural Burkina Faso
Source: PLoS One. 2022 Nov 17;17(11):e0277822. doi: 10.1371/journal.pone.0277822 (PMC9671424; doi:10.1371/journal.pone.0277822)
Supplement: S1 Checklist — (DOCX) [file pone.0277822.s001.docx]

# S1 Text. Additional quotes on barriers to secondary school

*“There are other illnesses that lead kids to be absent in school. (…) Often there is malaria and headaches which cause that a child is not there.” –* A, Teacher, male, 32 years

*“(…) When a girl is pregnant the fact is that coming to school the others will see the pregnancy. This is something the girl does not want and is a possible reason for her to abandon school.” –* A, Teacher, male, 32 years

*“(…) often there are others [students] that do not have a bicycle or those who have bicycles which often can have a glitch on the road, leading to [school] absence.” –* A, Teacher, male, 32 years

*“They [the administration] sent a part of the documents. What they sent us didn’t include the math materials. The materials for science classes are excluded. Only the materials for literature classes were sent. [When it was sent] it came last minute and in insufficient numbers and not for all subjects.” –* B, Teacher, male, 35 years

*“There are always problems. In some classrooms some windows are defect due to moisture and can’t be closed. Most doors don’t have keys (…). It’s the parents who buy the necessary furniture for their kids. –* D, Teacher, female, age unknown

*“We take water from barrels which can be stored for one week. After that, the water gets sticky and not drinkable.” –* K, Out-of-school adolescent, male, age unknown

*“[With security personnel] nothing can harm you. That could encourage the student to go to school”.* – C, Teacher, female, 26 years

*“On the axis Djibasso, the frontier to Mali, we are exposed every time when there are attacks. We are not spared. There is no security.”-* D, Teacher, female, age unknown

*“At the moment there are the jihadists. You never know!”* – G, Student, female, 20 years
