# Supplementary material for: “It’s the poverty”—Stakeholder perspectives on barriers to secondary education in rural Burkina Faso
Source: PLoS One. 2022 Nov 17;17(11):e0277822. doi: 10.1371/journal.pone.0277822 (PMC9671424; doi:10.1371/journal.pone.0277822)
Supplement: S1 File — (PDF) [file pone.0277822.s003.pdf]

Perceptions relatives à la santé et l'école secondaire : une étude utilisant des méthodes mixtes au Burkina Faso

(1) Questionnaire : Version pour les élèves inscrits à l'école

Pour commencer, laissez-moi vous remercier de parler avec moi aujourd'hui. Permettez-moi également de dire que pour les questions que je vais vous poser, il n'y a pas de bonnes ou de mauvaises réponses à mes questions. Je suis ici pour apprendre de vous à cause de vos expériences et de vos points de vue sur la scolarisation au Burkina Faso. Je suis ici pour recueillir des informations auprès de vous afin que d'autres (au Burkina Faso, en Afrique subsaharienne et dans le monde) puissent en apprendre davantage sur vos expériences et qu'ils puissent utiliser ces informations pour réaliser ou modifier d'autres programmes scolaires ou politiques dans des contextes similaires. Tout ce que vous dites sera anonymisé, ce qui signifie que personne ne pourra savoir qui a fourni cette information plus tard.

A) Introduction

1) **Racontez-moi un peu sur vos expériences à l'école :**
    a. Tu aimes bien aller à l'école ?
    b. Tu crois que c'est important pour mener une bonne vie / pour avoir du succès ?
2) **Pourrais-tu me raconter de ton environnement familial ?**
    a. Par rapport à la situation financière de votre famille...
    b. Comment sont les facilités et l'infrastructure à ton domicile ? (dans le contexte scolaire : bureau, endroit calme, électricité etc. disponible ?)
    c. As-tu recu de l'aide de ton entourage (tes parents / tes frères et soeurs / tes amis) pour tes devoirs ?
    d. Es-tu dépendant de ta famille ? Jusqu'à quel niveau/ dans quels domaines ? (finances, ...)
    e. Tes frères et soeurs, vont-ils à l'école ?

B) L'école à présent

3) **Raconte-moi une journée scolaire typique**
    a. Tu te lèves à quelle heure ?
    b. Comment arrives-tu à l'école ?
        i. Transport : à pied, byciclette, ...
        ii. Combien de temps dure le trajet ?
        iii. A-t-il des moments quand c'est plus difficile d'aller à l'école que normalement ? (par exemple pendant la saison de pluies, à cause d'une insécurité dans la région, ...)
    c. A-t-il le déjeuner à l'école ? (payant, pas payant ?)
    d. Tu finis les cours à quelle heure ? Tu rentres à la maison à quelle heure ?
    e. Dois-tu faire les devoirs après ? Quand finisses-tu tes devoirs ?
    f. Quelles sont tes taches à la maison ? À quelle heure vas-tu te coucher ?
4) **Qu'est-ce que tu en penses de l'infrastructure de ton école ?**
    a. Est-ce qu'il y a des latrines ? Sont-ils séparées (homme / femme) ? S'ils ne sont pas séparées : C'est un problème pour toi ? Pourquoi ?
    b. Est-ce qu'il y a de l'eau à l'école ? Est-il potable ?
    c. Combien d'élèves êtes-vous par par classe ? (environ)

(1) Questionnaire : Version pour les élèves inscrits à l'école

5) **Frais scolaires / Matériel pour l'école**

a. Est-ce que les matériaux nécessaires pour les cours sont fournis par l'école ? (par exemple : cahiers, crayons, uniforme scolaire...)
b. Est-ce qu'il faut acheter des matériaux ? C'est environ quel prix/ coût par mois ?
c. C'est qui qui paye les frais scolaires ? Les dépenses supplémentaires (par exemple repas pendant la journée, ...) ?
d. Est-ce qu'il est arrivé que tu as payé les frais d'une autre manière ? (par exemple en vendant du tabac, ou un comportement risqué) ?

<u>C) Obstacles</u>

6) **Obstacles**

a. Est-ce difficile pour vous d'aller à l'école / rester à l'école ? Pourquoi ?
b. Pourquoi est-ce difficile d'aller à l'école pour certains de vos amis ?
c. Tu te sens poussé à rester ou à quitter l'école ? De qui ? Dans quel sens ?
d. Quand était-il le plus difficile de rester à l'école ? Dans la transition ...
   i. Du primaire au collège
   ii. Du collège au lycée
b. Pourquoi était-il difficile de rester / continuer aller à l'école en ce moment ?

Sondes :

1. *Économique*
   - Coûts directs (frais de scolarité secondaire, frais d'appoint non réglementés etc.)
   - Coûts indirects (fournitures, déplacements, coûts d'opportunité, etc.)
2. *Socio-culturelle*
   - Biais de genre : mariages précoces
   - Peer-effets ou d'autres effets de réseau
3. *Psychologique*
   - Besoin perçu, qualité et rendement de la scolarité chez les enfants / parents
   - Redoublement : anxiété
4. *Santé*
   - Santé sexuelle et reproductive : grossesses précoces
   - Santé parentale ; sécurité alimentaire des ménages
5. *Structurel*
   - Distance à l'école secondaire
   - Barrières linguistiques : manque d'infrastructure (p.ex. toilettes séparés pour les filles)
   - Calendrier scolaire inapproprié

(1) Questionnaire : Version pour les élèves inscrits à l'école

6. *Politique et légal*
   - Les lois sur l'éducation obligatoire ; lois sur le travail des enfants
   - Age légal du mariage (17 et dans certains cas 15)
7. *Sécurité*
   - Voyage dangereux à l'école
   - Violence à l'école (pairs, attaques pat des groupes militants)
8. *Géographique*
   - Climat ; saison pluvieuse vs. saison sèche
9. *Historique*
   - Ségrégation

D) Interventions possibles

7) **Qu'est-ce qui rendrait plus facile pour toi / tes amis d'aller à l'école ?**
   a. Une moindre distance à l'école ?
   b. Des frais moins élevés pour l'école ? Abaisser les dépenses supplémentaires ? (Par exemple par transport gratuit, uniformes gratuits, etc.)
   c. Un programme de bourses d'études ? Combien de soutien financier serait nécessaire ?
   d. Transferts en espèces à toi / à tes parents (conditionnels pour aller à l'école) ?
   e. Personnel de sécurité à l'école / sur le chemin de l'école ?
   f. Changement de perception des avantages pour l'école ?
   g. As-tu une proposition ?

E) L'école et la santé

8) **Penses-tu que l'école protège des problèmes de santé suivants à court terme (déjà aujourd'hui, cette année) ?**
   a. Santé sexuelle et reproductive, grossesses jeunes, mariages jeunes
   b. Risque d'infection VIH
   c. Comportement risqué : alcool, drogues, ...
   d. Santé mentale, violence et blessures
   e. Accès au système de soins, assurance-maladie
   f. Normes et attitudes
9) **Pensez-vous que l'école protège des problèmes de santé suivants à long terme (dans environ dix ans) ?**
   a. Santé sexuelle et reproductive, grossesses jeunes, mariages jeunes
   b. Risque d'infection VIH
   c. Comportement risqué : alcool, drogues, ...
   d. Santé mentale, violence et blessures
   e. Accès au système de soins, assurance-maladie
   f. Salaires augmentés, opportunités d'emploi améliorés
   g. Normes et attitudes

(1) Questionnaire : Version pour les élèves inscrits à l'école

Suggestions si l'informateur ne sait pas comment l'éducation et la santé peuvent être liées : "Peut-être que le fait d'aller à l'école influence d'une manière que ..."

- Tu passes moins de temps dans la rue -> moins d'occasions de fumer ou de boire de l'alcool ?
- L'école t'as informé sur certains sujets afin que tu aie maintenant un comportement plus sain / moins risqué (nutrition, alcool, tabac, utilisation de préservatifs, ..) ?
- Tu as trouvé des amis ou des modèles qui donnent un bon exemple ?
- Cela t'aide à avoir un meilleur travail / de meilleurs salaires à l'avenir et donc facilite-t-il la vie autonome / l'achat de médicaments ?

10) Parlons-nous de vos interactions avec le système de soins

a. A une scolarité supplémentaire t'aidé à mieux comprendre savoir médical ?
   - Messages de santé publique
   - Prescriptions

b. Comment a la scolarité supplémentaire influencée tes interactions avec le système de soins et le personnel de la santé ?
   - A-t-il facilité la communication ?
   - A-t-il amélioré les résultats de santé ?
   - A-t-il amélioré d'autre paramètres (p.e. social, comportemental, économique) ?

c. A la scolarité facilité l'interaction de tes membres de famille avec le système de soins ?
   - Tes parents ?

F) Effets de débordement

11) Partages-tu ces bénéfices avec ton entourage ?

a. Parles-tu de ce que tu as appris à l'école avec ton entourage ?
b. Si oui - avec qui ? Frères et sœurs ? Parents ? Autres ?
c. Partages-tu ton revenu avec ton entourage ?
d. Si oui - avec qui ? Frères et sœurs ? Parents ? Autres ?

G) Attentes et plans futurs

12) Continuité de l'école :

a. Quel âge avais-tu lorsque tu as commencé l'école?
b. Es-tu allé continuellement ? (Répétitions, abandons ?) Si tu n'es pas allé continuellement, pourquoi ?

13) Qui décide de la scolarité ?

a. Qui t'a inscrit à l'école ? (parent, chef de tribu / autre autorité?) Voulais-tu y aller toi-même ?
b. Qui voulait que tu continues aller à l'école ?
c. Qui pousse pour et qui s'oppose à aller à l'école ?

(1) Questionnaire : Version pour les élèves inscrits à l'école

14) **Combien de temps espéres / prévois-tu de rester à l'école ?**
   a. À quelles écoles espéres-tu aller à l'avenir et jusqu'où sont-elles à la maison ?

15) **Qu'aimerais-tu faire plus tard dans la vie ? / Quels sont tes espoirs pour l'avenir ?**
   a. En général ? Travail et carrière ? Parent à la maison ?
   b. Quel travail ? (Si le travail)
   c. Pensez-vous que vous trouverez facilement un emploi après l'obtention du diplôme ? (c'est-à-dire, les opportunités sur le marché du travail). Si oui, quel genre de travail ? À la maison ou salarié ?

16) **Quelles sont les attentes de tes parents concernant votre scolarité ?**

17) **Qu'attendez-vous d'aller à l'école ?**
   (avantages généraux et spécifiques) :
   a. Avantages
   b. Monétaire / non monétaire : respect, succès, ...
   c. Résultats de santé
   ... pour vous-même, pour les autres, par ex. famille : parents, ...

H) Fin

18) **Y a-t-il quelque chose que je ne vous ai pas demandé et que j'aurais dû vous demander ?**
   a. *Si le répondant dit quelque chose, posez cette question*

19) **Y a-t-il autre chose que vous aimeriez ajouter ?**

20) **Je cherche à rassembler autant d'informations que possible sur l'expérience burkinabè en termes d'accès et de bénéfices perçus pour l'école. Avec qui d'autre me recommanderiez-vous parler ? S'il vous plaît donnez-moi l'orthographe et si possible des informations de contact pour cette personne**.

Perceptions relatives à la santé et l'école secondaire : une étude utilisant des méthodes mixtes au Burkina Faso

(1b) Questionnaire : Version pour les élèves inscrits au lycée

Pour commencer, laissez-moi vous remercier de parler avec moi aujourd'hui. Permettez-moi également de dire que pour les questions que je vais vous poser, il n'y a pas de bonnes ou de mauvaises réponses à mes questions. Je suis ici pour apprendre de vous à cause de vos expériences et de vos points de vue sur la scolarisation au Burkina Faso. Je suis ici pour recueillir des informations auprès de vous afin que d'autres (au Burkina Faso, en Afrique subsaharienne et dans le monde) puissent en apprendre davantage sur vos expériences et qu'ils puissent utiliser ces informations pour réaliser ou modifier d'autres programmes scolaires ou politiques dans des contextes similaires. Tout ce que vous dites sera anonymisé, ce qui signifie que personne ne pourra savoir qui a fourni cette information plus tard.

A) Introduction

1) **Racontez-moi un peu sur vos expériences à l'école :**
    a. Tu aimes bien aller à l'école ?
    b. Tu crois que c'est important pour mener une bonne vie / pour avoir du succès ?
2) **Pourrais-tu me raconter de ton environnement familial ?**
    a. Par rapport à la situation financière de votre famille...
    b. Comment sont les facilités et l'infrastructure à ton domicile ? (dans le contexte scolaire : bureau, endroit calme, électricité etc. disponible ?)
    c. As-tu recu de l'aide de ton entourage (tes parents / tes frères et soeurs / tes amis) pour tes devoirs ?
    d. Es-tu dépendant de ta famille ? Jusqu'à quel niveau/ dans quels domaines ? (finances, ...)
    e. Tes frères et soeurs, vont-ils à l'école ?

B) L'école à présent

3) **Raconte-moi une journée scolaire typique**
    a. Tu te lèves à quelle heure ?
    b. Comment arrives-tu à l'école ?
        i. Transport : à pied, byciclette, ...
        ii. Combien de temps dure le trajet ?
        iii. A-t-il des moments quand c'est plus difficile d'aller à l'école que normalement ? (par exemple pendant la saison de pluies, à cause d'une insécurité dans la région, ...)
    c. A-t-il le déjeuner à l'école ? (payant, pas payant ?)
    d. Tu finis les cours à quelle heure ? Tu rentres à la maison à quelle heure ?
    e. Dois-tu faire les devoirs après ? Quand finisses-tu tes devoirs ?
    f. Quelles sont tes taches à la maison ? À quelle heure vas-tu te coucher ?
4) **Qu'est-ce que tu en penses de l'infrastructure de ton école ?**
    a. Est-ce qu'il y a des latrines ? Sont-ils séparées (homme / femme) ? S'ils ne sont pas séparées : C'est un problème pour toi ? Pourquoi ?
    b. Est-ce qu'il y a de l'eau à l'école ? Est-il potable ?
    c. Combien d'élèves êtes-vous par par classe ? (environ)

(1b) Questionnaire : Version pour les élèves inscrits au lycée

5) **Frais scolaires / Matériel pour l'école**
   a. Est-ce que les matériaux nécessaires pour les cours sont fournis par l'école ? (par exemple : cahiers, crayons, uniforme scolaire...)
   b. Est-ce qu'il faut acheter des matériaux ? C'est environ quel prix/ coût par mois ?
   c. C'est qui qui paye les frais scolaires ? Les dépenses supplémentaires (par exemple repas pendant la journée, ...) ?
   d. Est-ce qu'il est arrivé que tu as payé les frais d'une autre manière ? (par exemple en vendant du tabac, ou un comportement risqué) ?

C) Obstacles

6) **Obstacles**
   a. Est-ce difficile pour vous d'aller à l'école / rester à l'école ? Pourquoi ?
   b. Quand était-il le plus difficile de rester à l'école ? Dans la transition ...
      i. Du primaire au collège
      ii. Du collège au lycée
   b. Pourquoi était-il difficile de rester / continuer aller à l'école en ce moment ?
7) **Est-ce que tu as des amis qui n'ont pas pu poursuivre jusqu'au lycée ? Quels étaient les raisons ?**

Sondes :

1. *Économique*
   - Coûts directs (frais de scolarité secondaire, frais d'appoint non réglementés etc.)
   - Coûts indirects (fournitures, déplacements, coûts d'opportunité, etc.)
2. *Socio-culturelle*
   - Biais de genre : mariages précoces
   - Peer-effets ou d'autres effets de réseau
3. *Psychologique*
   - Besoin perçu, qualité et rendement de la scolarité chez les enfants / parents
   - Redoublement : anxiété
4. *Santé*
   - Santé sexuelle et reproductive : grossesses précoces
   - Santé parentale ; sécurité alimentaire des ménages
5. *Structurel*
   - Distance à l'école secondaire
   - Barrières linguistiques : manque d'infrastructure (p.ex. toilettes séparés pour les filles)
   - Calendrier scolaire inapproprié

6. *Politique et légal*
   - Les lois sur l'éducation obligatoire ; lois sur le travail des enfants
   - Age légal du mariage (17 et dans certains cas 15)
7. *Sécurité*
   - Voyage dangereux à l'école
   - Violence à l'école (pairs, attaques pat des groupes militants)
8. *Géographique*
   - Climat ; saison pluvieuse vs. saison sèche
9. *Historique*
   - Ségrégation

D) Interventions possibles

7) **Qu'est-ce qui rendrait plus facile pour toi / tes amis d'aller à l'école ?**
   a. Une moindre distance à l'école ?
   b. Des frais moins élevés pour l'école ? Abaisser les dépenses supplémentaires ? (Par exemple par transport gratuit, uniformes gratuits, etc.)
   c. Un programme de bourses d'études ? Combien de soutien financier serait nécessaire ?
   d. Transferts en espèces à toi / à tes parents (conditionnels pour aller à l'école) ?
   e. Personnel de sécurité à l'école / sur le chemin de l'école ?
   f. Changement de perception des avantages pour l'école ?
   g. As-tu une proposition ?

E) L'école et la santé

8) **Penses-tu que l'école protège des problèmes de santé suivants à court terme (déjà aujourd'hui, cette année) ?**
   a. Santé sexuelle et reproductive, grossesses jeunes, mariages jeunes
   b. Risque d'infection VIH
   c. Comportement risqué : alcool, drogues, ...
   d. Santé mentale, violence et blessures
   e. Accès au système de soins, assurance-maladie
   f. Normes et attitudes
9) **Pensez-vous que l'école protège des problèmes de santé suivants à long terme (dans environ dix ans) ?**
   a. Santé sexuelle et reproductive, grossesses jeunes, mariages jeunes
   b. Risque d'infection VIH
   c. Comportement risqué : alcool, drogues, ...
   d. Santé mentale, violence et blessures
   e. Accès au système de soins, assurance-maladie
   f. Salaires augmentés, opportunités d'emploi améliorés
   g. Normes et attitudes

(1b) Questionnaire : Version pour les élèves inscrits au lycée

Suggestions si l'informateur ne sait pas comment l'éducation et la santé peuvent être liées : "Peut-être que le fait d'aller à l'école influence d'une manière que ..."

- Tu passes moins de temps dans la rue -> moins d'occasions de fumer ou de boire de l'alcool ?
- L'école t'as informé sur certains sujets afin que tu aie maintenant un comportement plus sain / moins risqué (nutrition, alcool, tabac, utilisation de préservatifs, ..) ?
- Tu as trouvé des amis ou des modèles qui donnent un bon exemple ?
- Cela t'aide à avoir un meilleur travail / de meilleurs salaires à l'avenir et donc facilite-t-il la vie autonome / l'achat de médicaments ?

10) Parlons-nous de vos interactions avec le système de soins

a. A une scolarité supplémentaire t'aidé à mieux comprendre savoir médical ?
    - Messages de santé publique
    - Prescriptions
b. Comment a la scolarité supplémentaire influencée tes interactions avec le système de soins et le personnel de la santé ?
    - A-t-il facilité la communication ?
    - A-t-il amélioré les résultats de santé ?
    - A-t-il amélioré d'autre paramètres (p.e. social, comportemental, économique) ?
c. A la scolarité facilité l'interaction de tes membres de famille avec le système de soins ?
    - Tes parents ?

F) Effets de débordement

11) Partages-tu ces bénéfices avec ton entourage ?

a. Parles-tu de ce que tu as appris à l'école avec ton entourage ?
b. Si oui - avec qui ? Frères et sœurs ? Parents ? Autres ?
c. Partages-tu ton revenu avec ton entourage ?
d. Si oui - avec qui ? Frères et sœurs ? Parents ? Autres ?

G) Attentes et plans futurs

12) Continuité de l'école :

a. Quel âge avais-tu lorsque tu as commencé l'école?
b. Es-tu allé continuellement ? (Répétitions, abandons ?) Si tu n'es pas allé continuellement, pourquoi ?

13) Qui décide de la scolarité ?

a. Qui t'a inscrit à l'école ? (parent, chef de tribu / autre autorité?) Voulais-tu y aller toi-même ?
b. Qui voulait que tu continues aller à l'école ?
c. Qui pousse pour et qui s'oppose à aller à l'école ?

(1b) Questionnaire : Version pour les élèves inscrits au lycée

14) **Combien de temps espéres / prévois-tu de rester à l'école ?**
    a. À quelles écoles espéres-tu aller à l'avenir et jusqu'où sont-elles à la maison ?
15) **Qu'aimerais-tu faire plus tard dans la vie ? / Quels sont tes espoirs pour l'avenir ?**
    a. En général ? Travail et carrière ? Parent à la maison ?
    b. Quel travail ? (Si le travail)
    c. Pensez-vous que vous trouverez facilement un emploi après l'obtention du diplôme ? (c'est-à-dire, les opportunités sur le marché du travail). Si oui, quel genre de travail ? À la maison ou salarié ?
16) **Quelles sont les attentes de tes parents concernant votre scolarité ?**
17) **Qu'attendez-vous d'aller à l'école ?**
    (avantages généraux et spécifiques) :
    a. Avantages
    b. Monétaire / non monétaire : respect, succès, ...
    c. Résultats de santé
       ... pour vous-même, pour les autres, par ex. famille : parents, ...

H) Fin

18) **Y a-t-il quelque chose que je ne vous ai pas demandé et que j'aurais dû vous demander ?**
    a. *Si le répondant dit quelque chose, posez cette question*
19) **Y a-t-il autre chose que vous aimeriez ajouter ?**
20) **Je cherche à rassembler autant d'informations que possible sur l'expérience burkinabè en termes d'accès et de bénéfices perçus pour l'école. Avec qui d'autre me recommanderiez-vous parler ? S'il vous plaît donnez-moi l'orthographe et si possible des informations de contact pour cette personne**.

(2) Questionnaire : Version pour les élèves ayant abandonné l'école

Pour commencer, laissez-moi vous remercier de parler avec moi aujourd'hui. Permettez-moi également de dire que pour les questions que je vais vous poser, il n'y a pas de bonnes ou de mauvaises réponses à mes questions. Je suis ici pour apprendre de vous à cause de vos expériences et de vos points de vue sur la scolarisation au Burkina Faso. Je suis ici pour recueillir des informations auprès de vous afin que d'autres (au Burkina Faso, en Afrique subsaharienne et dans le monde) puissent en apprendre davantage sur vos expériences et qu'ils puissent utiliser ces informations pour réaliser ou modifier d'autres programmes scolaires ou politiques dans des contextes similaires. Tout ce que vous dites sera anonymisé, ce qui signifie que personne ne pourra savoir qui a fourni cette information plus tard.

A) Introduction

1) Qu'est-ce que tu fais dans la vie ? Qu'est-ce que tu faisais avant/ tu vas faire dans le futur ?
    a. Es-tu employé/salarié/occupé ? Légalement/Illégalement ?
    b. De quoi tu travailles ?

2) **Pourrais-tu me raconter de ton environnement familial ?**
    a. Par rapport à la situation financière de ta famille...
    b. Comment sont les facilités et l'infrastructure à ton domicile ? (Dans le contexte scolaire : bureau, endroit calme, électricité etc. disponible ?)
    c. As-tu reçu de l'aide de ton entourage (tes parents / tes frères et sœurs / tes amis) pour tes devoirs ?
    d. Tu es dépendant de ta famille ? Jusqu'à quel niveau/ dans quels domaines ? (Finances, ...)
    e. Tes frères et sœurs, vont-ils à l'école ?

B) Situation dans l'ancienne école

3) **Raconte-moi un peu sur tes expériences à l'école :**
    a. Tu aimais bien aller à l'école ?
    b. Ça fait combien de temps que tu as abandonné l'école ?
    c. Tu crois que c'est important pour mener une bonne vie / pour avoir du succès ?

4) **Raconte-moi une journée scolaire typique**
    a. Tu te levais à quelle heure ?
    b. Comment arrivais-tu à l'école ?
        i. Transport : à pied, bicyclette ...
        ii. Combien de temps durait le trajet ?
        iii. A-t-il des moments quand c'était plus difficile d'aller à l'école que normalement ? (P.ex. pendant la saison de pluies, à cause d'une insécurité dans la région, ...)
    c. Avait-t-il le déjeuner à l'école ? (Payant, pas payant ?)
    d. Tu finissais les cours à quelle heure ? Tu rentrais à la maison à quelle heure ?

(2) Questionnaire : Version pour les élèves ayant abandonné l'école

   e. Devais-tu faire les devoirs après ? Quand finissais-tu tes devoirs ?
   f. Quelles étaient tes taches à la maison ? À quelle heure allais-tu te coucher ?

5) **Qu'est-ce que tu en penses de l'infrastructure de ton ancienne école ?**
   a. Est-ce qu'il y avait des latrines ? Étaient-ils séparés (homme / femme) ? S'ils n'étaient pas séparés : C'est un problème pour toi ? Pourquoi ?
   b. Est-ce qu'il y avait de l'eau à l'école ? Était-il potable ?
   c. Combien d'élèves étaient-vous par classe ? (environ)

6) Qui décide de la scolarité ?
   a. Qui t'a inscrit à l'école ? (parent, chef de tribu / autre autorité?) Voulais-tu y aller toi-même ?
   b. Qui voulait que tu continues aller à l'école ?
   c. Qui était pour ou contre d'aller à l'école ?

7) Continuité de l'école :
   a. Quel âge avais-tu lorsque tu as commencé l'école ?
   b. Es-tu allé continuellement ? (Répétitions, abandons ?) Si tu n'es pas allé continuellement, pourquoi ?

C) Obstacles

8) Pourquoi as-tu abandonné l'école ?
   a. Quelle était la raison principale pour laquelle tu as abandonné ?
   b. Est-ce que quelqu'un était en colère ou triste parce que tu as abandonné ?
   c. Tes parents ont-ils soutenu cette décision ? Tes amis ?
   d. Dans quelle classe as tu quitté l'école ?
   e. Pourquoi était-il difficile de rester à l'école en ce moment du passé ?

Sondes :

1. *Économique*
   - Coûts directs (frais de scolarité secondaire, frais d'appoint non réglementés etc.)
   - Coûts indirects (fournitures, déplacements, coûts d'opportunité, etc.)
2. *Socio-culturelle*
   - Biais de genre : mariages précoces
   - Peer-effets ou d'autres effets de réseau
3. *Psychologique*
   - Besoin perçu, qualité et rendement de la scolarité chez les enfants / parents
   - Redoublement : anxiété
4. *Santé*
   - Santé sexuelle et reproductive : grossesses précoces
   - Santé parentale ; sécurité alimentaire des ménages

(2) Questionnaire : Version pour les élèves ayant abandonné l'école

5. *Structurel*
   - Distance à l'école secondaire
   - Barrières linguistiques : manque d'infrastructure (p.ex. toilettes séparés pour les filles)
   - Calendrier scolaire inapproprié
6. *Politique et légal*
   - Les lois sur l'éducation obligatoire ; lois sur le travail des enfants
   - Age légal du mariage (17 et dans certains cas 15)
7. *Sécurité*
   - Voyage dangereux à l'école
   - Violence à l'école (pairs, attaques pat des groupes militants)
8. *Géographique*
   - Climat ; saison pluvieuse vs. saison sèche
9. *Historique*
   - Ségrégation

D) Interventions possibles

9) **Qu'est-ce qui rendrait plus facile pour toi / tes amis d'aller à l'école ?**

   a. Une moindre distance à l'école ?
   b. Des frais moins élevés pour l'école ? Abaisser les dépenses supplémentaires ? (Par exemple par transport gratuit, uniformes gratuits, etc.)
   c. Un programme de bourses d'études ? Combien de soutien financier serait nécessaire ?
   d. Transferts en espèces à toi / à tes parents (conditionnels pour aller à l'école) ?
   e. Personnel de sécurité à l'école / sur le chemin de l'école ?
   f. Changement de perception des avantages pour l'école ?
   g. As-tu une proposition ?

E) L'école et la santé

10) **Penses-tu que l'école protège des problèmes de santé suivants à court terme (déjà aujourd'hui, cette année) ?**
   a. Santé sexuelle et reproductive, grossesses jeunes, mariages jeunes
   b. Risque d'infection VIH
   c. Comportement risqué : alcool, drogues, ...
   d. Santé mentale, violence et blessures
   e. Accès au système de soins, assurance-maladie
   f. Normes et attitudes

11) **Penses-tu que l'école protège des problèmes de santé suivants à long terme (dans environ dix ans) ?**
   a. Santé sexuelle et reproductive, grossesses jeunes, mariages jeunes

(2) Questionnaire : Version pour les élèves ayant abandonné l'école

b. Risque d'infection VIH
c. Comportement risqué : alcool, drogues, ...
d. Santé mentale, violence et blessures
e. Accès au système de soins, assurance-maladie
f. Salaires augmentés, opportunités d'emploi améliorés
g. Normes et attitudes

Suggestions si l'informateur ne sait pas comment l'éducation et la santé peuvent être liées : "Peut-être que le fait d'aller à l'école influence d'une manière que ..."

- Tu passes moins de temps dans la rue -> moins d'occasions de fumer ou de boire de l'alcool ?
- L'école t'as informé sur certains sujets afin que tu aie maintenant un comportement plus sain / moins risqué (nutrition, alcool, tabac, utilisation de préservatifs, ..) ?
- Tu as trouvé des amis ou des modèles qui donnent un bon exemple ?
- Cela t'aide-t-il à avoir un meilleur travail / de meilleurs salaires à l'avenir et donc facilite-t-il la vie autonome / l'achat de médicaments ?

12) **Parlons-nous de vos interactions avec le système de soins**

a. A une scolarité supplémentaire t'aidé à mieux comprendre savoir médical ?
   - o Messages de santé publique
   - o Prescriptions

b. Comment a la scolarité supplémentaire influencée tes interactions avec le système de soins et le personnel de la santé ?
   - o A-t-il facilité la communication ?
   - o A-t-il amélioré les résultats de santé ?
   - o A-t-il amélioré d'autre paramètres (p.e. social, comportemental, économique) ?

c. A la scolarité facilité l'interaction de tes membres de famille avec le système de soins ?
   - o Tes parents ?

F) Effets de débordement

13) **Partages-tu ces bénéfices avec ton entourage ?**

a. Parles-tu de ce que tu as appris à l'école avec ton entourage ?
b. Si oui - avec qui ? Frères et sœurs ? Parents ? Autres ?
c. Partages-tu ton revenu avec ton entourage ?
d. Si oui - avec qui ? Frères et sœurs ? Parents ? Autres ?

G) Attentes et plans futurs

14) **Penses-tu reprendre l'école ? Aimerais-tu reprendre l'école ?**

15) **Quelles sont les attentes de tes parents ?**

a. Est-ce une scolarité nécessaire pour cela ?

(2) Questionnaire : Version pour les élèves ayant abandonné l’école

H) Fin

16) **Y a-t-il quelque chose que je ne t’ai pas demandé et que j'aurais dû te demander ?**
    a. *Si le répondant dit quelque chose, posez cette question*
17) **Y a-t-il autre chose que tu aimeriez ajouter ?**
18) **Je cherche à rassembler autant d'informations que possible sur l'expérience burkinabè en termes d'accès et de bénéfices perçus pour l'école. Avec qui d'autre me recommanderiez-tu parler ? S'il tu plaît donnez-moi l'orthographe et si possible des informations de contact pour cette personne**.

(3) Questionnaire : Version pour les parents

Pour commencer, laissez-moi vous remercier de parler avec moi aujourd'hui. Permettez-moi également de dire que pour les questions que je vais vous poser, il n'y a pas de bonnes ou de mauvaises réponses à mes questions. Je suis ici pour apprendre de vous à cause de vos expériences et de vos points de vue sur la scolarisation au Burkina Faso. Je suis ici pour recueillir des informations auprès de vous afin que d'autres (au Burkina Faso, en Afrique subsaharienne et dans le monde) puissent en apprendre davantage sur vos expériences et qu'ils puissent utiliser ces informations pour réaliser ou modifier d'autres programmes scolaires ou politiques dans des contextes similaires. Tout ce que vous dites sera anonymisé, ce qui signifie que personne ne pourra savoir qui a fourni cette information plus tard.

A) Introduction / Contexte

1) **Parlez-moi un peu de vous:**
   une.
   - **a.** Où habitez-vous (isolé / distance de l'école et d'autres installations?)?
2) **Parlez-moi un peu de vos expériences avec l'école:**
   - **a.** Êtes-vous allé à l'école?
   - **b.** Combien d'années?
   - **c.** Pourquoi avez-vous arrêté d'aller à l'école?
3) **De quelle famille venez-vous?**
   - **a.** Éducation des parents, emploi, situation financière, situation géographique, langue?
4) **Comment votre expérience scolaire a-t-elle influencé votre point de vue sur vos enfants qui vont à l'école?**

B) Obstacles

5) **Que devez-vous fournir pour envoyer votre enfant à l'école?**
   - **a.** Combien coûte-t-il ?
     - **i.** frais de scolarité par an
     - **ii.** dépenses supplémentaires (p. ex. bouqins etc.) par mois
   - **b.** Est-ce que vous vous attendez à ce que vos enfants contribuent à cela?
   - **c.** Est-il difficile d'obtenir l'argent nécessaire pour l'école ?
6) **Quelle est la plus grande partie / la plus difficile?**
7) *Si l'enfant ne va plus à l'école:* **Pourquoi votre enfant a-t-il abandonné l'école?**
8) **Quels sont / étaient les obstacles pour aller à l'école?**
9) **Quand a-t-il été plus difficile de continuer à envoyer votre enfant à l'école?**
   1. Du primaire au collège
   2. Du collège au lycée
10) **Pourquoi était-il plus difficile de continuer à envoyer votre enfant à l'école?**

(3) Questionnaire : Version pour les parents

Sondes

1. *Économique*
    - Coûts directs (frais de scolarité secondaire, frais d'appoint non réglementés etc.)
    - Coûts indirects (fournitures, déplacements, coûts d'opportunité, etc.)
2. *Socio-culturelle*
    - Biais de genre : mariages précoces
    - Peer-effets ou d'autres effets de réseau
3. *Psychologique*
    - Besoin perçu, qualité et rendement de la scolarité chez les enfants / parents
    - Redoublement : anxiété
4. *Santé*
    - Santé sexuelle et reproductive : grossesses précoces
    - Santé parentale ; sécurité alimentaire des ménages
5. *Structurel*
    - Distance à l'école secondaire
    - Barrières linguistiques : manque d'infrastructure (p.ex. toilettes séparés pour les filles)
    - Calendrier scolaire inapproprié
6. *Politique et légal*
    - Les lois sur l'éducation obligatoire ; lois sur le travail des enfants
    - Age légal du mariage (17 et dans certains cas 15)
7. *Sécurité*
    - Voyage dangereux à l'école
    - Violence à l'école (pairs, attaques pat des groupes militants)
8. *Géographique*
    - Climat ; saison pluvieuse vs. saison sèche
9. *Historique*
    - Ségrégation

C) <u>Interventions possibles</u>

11) **Qu'est-ce qui rendrait plus facile envoyer votre enfant à l'école ?**

a. Une moindre distance à l'école ?

b. Des frais moins élevés pour l'école ? Abaisser les dépenses supplémentaires ? (Par exemple par transport gratuit, uniformes gratuits, etc.)

c. Un programme de bourses d'études ? Combien de soutien financier serait nécessaire ?

d. Transferts en espèces à vous / votre enfant (conditionnels pour aller à l'école) ?

e. Personnel de sécurité à l'école / sur le chemin de l'école ?
f. Changement de perception des avantages pour l'école ?
g. Avez-vous une proposition ?

D) L’école et la santé

12) **Qu’est-ce que votre enfant apprend à l’école concernant la santé ?**
13) **Pensez-vous que l'école protège des problèmes de santé suivants à court terme (déjà aujourd'hui, cette année) ?**
    a. Santé sexuelle et reproductive, grossesses jeunes, mariages jeunes
    b. Risque d’infection VIH
    c. Comportement risqué : alcool, drogues, ...
    d. Santé mentale, violence et blessures
    e. Accès au système de soins, assurance-maladie
    f. Normes et attitudes
14) **Pensez-vous que l'école protège des problèmes de santé suivants à long terme (dans environ dix ans) ?**
    a. Santé sexuelle et reproductive, grossesses jeunes, mariages jeunes
    b. Risque d’infection VIH
    c. Comportement risqué : alcool, drogues, ...
    d. Santé mentale, violence et blessures
    e. Accès au système de soins, assurance-maladie
    f. Salaires augmentés, opportunités d’emploi améliorés
    g. Normes et attitudes

Suggestions si l'informateur ne sait pas comment l'éducation et la santé peuvent être liées : "Peut-être que le fait d'aller à l'école influence d'une manière que ..."

- Votre enfant passe moins de temps dans la rue -> moins d'occasions de fumer ou de boire de l'alcool ?
- L’école informe votre enfant sur certains sujets afin qu’il ait maintenant un comportement plus sain / moins risqué (nutrition, alcool, tabac, utilisation de préservatifs, ..) ?
- Il trouva des amis ou des modèles qui donnent un bon exemple ?
- Cela lui aide à avoir un meilleur travail / de meilleurs salaires à l'avenir et donc facilite la vie autonome / l'achat de médicaments ?

15) **Pensez-vous que votre enfant aurait des habitudes plus malsaines s'il n'était pas à l'école?** (Tabac, alcool, autres drogues)
16) **Pensez-vous que votre enfant se comporte de façon plus sage / moins risquée en raison de sa scolarité?**
17) **Parlons-nous de vos interactions avec le système de soins**
    a. A une scolarité supplémentaire t’aidé à mieux comprendre savoir médical ?
        i. Messages de santé publique
        ii. Prescriptions

(3) Questionnaire : Version pour les parents

b. Comment a la scolarité supplémentaire influencée tes interactions avec le système de soins et le personnel de la santé ?
   i. A-t-il facilité la communication ?
   ii. A-t-il amélioré les résultats de santé ?
   iii. A-t-il amélioré d'autre paramètres (p.e. social, comportemental, économique) ?

c. A votre enfant vous-a-t-il déjà aidé dans des interactions avec le système de santé ? Grâce à sa scolarité ?

E) Effets de débordement

18) **Est-ce que votre enfant partage les avantages acquis à l'école avec les gens autour d'eux ?**
   a. Si oui - avec qui ? Frères et sœurs ? Vous ? Autres ?
   b. Lesquels ? Connaissance ? Les salaires résultant de meilleurs emplois ?

F) Attentes

19) **Comment pensez-vous que votre enfant va bénéficier de l'école?**
   a. Travail dans le futur?
   b. Sera-t-il plus heureux / en meilleure santé / ...?
20) **Vous attendez-vous à ce qu'ils prennent soin de vous quand vous êtes vieux?**
21) **Quelles sont vos attentes d'envoyer vos enfants à l'école?**
   (avantages généraux et spécifiques):
   - avantages
   - monétaire
   - non monétaire: respect, succès, ...
   - résultats de santé
   - pour vous-même, pour les autres, par ex. famille: parents, ...)

   Quel niveau d'études pensez-vous essentiel pour atteindre ces objectifs?

22) **Avez-vous communiqué ces attentes à vos enfants?**

G) Fin

23) **Y a-t-il quelque chose que je ne vous ai pas demandé et que j'aurais dû vous demander ?**
   a. *Si le répondant dit quelque chose, posez cette question*
24) **Y a-t-il autre chose que vous aimeriez ajouter ?**
25) **Je cherche à rassembler autant d'informations que possible sur l'expérience burkinabè en termes d'accès et de bénéfices perçus pour l'école. Avec qui d'autre me recommanderiez-vous parler ? S'il vous plaît donnez-moi l'orthographe et si possible des informations de contact pour cette personne.**

Perceptions relatives à la santé et l'école secondaire : une étude utilisant des méthodes mixtes au Burkina Faso

(3b) Questionnaire : Version pour les parents avec enfant qui n'est pas en cours de scolarisation

Pour commencer, laissez-moi vous remercier de parler avec moi aujourd'hui. Permettez-moi également de dire que pour les questions que je vais vous poser, il n'y a pas de bonnes ou de mauvaises réponses à mes questions. Je suis ici pour apprendre de vous à cause de vos expériences et de vos points de vue sur la scolarisation au Burkina Faso. Je suis ici pour recueillir des informations auprès de vous afin que d'autres (au Burkina Faso, en Afrique subsaharienne et dans le monde) puissent en apprendre davantage sur vos expériences et qu'ils puissent utiliser ces informations pour réaliser ou modifier d'autres programmes scolaires ou politiques dans des contextes similaires. Tout ce que vous dites sera anonymisé, ce qui signifie que personne ne pourra savoir qui a fourni cette information plus tard.

A) Introduction / Contexte

1) **Parlez-moi un peu de vous :**
    a. Où habitez-vous (isolé / distance de l'école et d'autres installations...) ?
2) **Parlez-moi un peu de vos expériences avec l'école :**
    a. Pourquoi avez-vous arrêté d'aller à l'école ?
3) **De quelle famille venez-vous ?**
    a. Éducation des parents, emploi, situation financière
4) *Si le parent à eu une expérience scolaire :* **Comment votre expérience scolaire a-t-elle influencé votre point de vue sur la scolarisation de vos enfants ?**

B) Obstacles

5) **Considérez-vous important que votre enfant aille à l'école ?** (Pourquoi oui / Pourquoi non ?)
6) *Si l'enfant a abandonné l'école :* **Pourquoi votre enfant a-t-il abandonné l'école ?**
   Si l'enfant et jamais allé à l'école : **Pourquoi a votre enfant jamais fréquenté l'école ?**
7) **Que devriez-vous fournir pour envoyer votre enfant à l'école ?**
    a. Est-il difficile de fournir ca ?
    b. Quoi exactement ? (l'argent en espèces, les matériaux, ...)
    c. Pourquoi est-il difficile ?
8) **Quelle est la plus grande partie / la partie la plus difficile ?**
9) **Quels sont / étaient les obstacles pour aller à l'école ?**
10) **Est-ce que vous en avez besoin d'aide de votre enfant pour votre travail** (p.ex. cultiver le champ, participer dans le commerce,...)
11) **Qui décide sur la scolarisation de votre enfant ?**

Sondes

1. *Économique*
    - Coûts directs (frais de scolarité secondaire, frais d'appoint non réglementés etc.)
    - Coûts indirects (fournitures, déplacements, coûts d'opportunité, etc.)
2. *Socio-culturelle*
    - Biais de genre : mariages précoces

(3b) Questionnaire : Version pour les parents avec enfant qui n'est pas en cours de scolarisation

- Peer-effets ou d'autres effets de réseau

3. *Psychologique*
   - Besoin perçu, qualité et rendement de la scolarité chez les enfants / parents
   - Redoublement : anxiété
4. *Santé*
   - Santé sexuelle et reproductive : grossesses précoces
   - Santé parentale ; sécurité alimentaire des ménages
5. *Structurel*
   - Distance à l'école secondaire
   - Barrières linguistiques : manque d'infrastructure (p.ex. toilettes séparés pour les filles)
   - Calendrier scolaire inapproprié
6. *Politique et légal*
   - Les lois sur l'éducation obligatoire ; lois sur le travail des enfants
   - Age légal du mariage (17 et dans certains cas 15)
7. *Sécurité*
   - Voyage dangereux à l'école
   - Violence à l'école (pairs, attaques pat des groupes militants)
8. *Géographique*
   - Climat ; saison pluvieuse vs. saison sèche
9. *Historique*
   - Ségrégation

C) Interventions possibles

12) **Qu'est-ce qui rendrait plus facile envoyer votre enfant à l'école ?**

a. Une moindre distance à l'école ?

b. Des frais moins élevés pour l'école ? Abaisser les dépenses supplémentaires ? (Par exemple par transport gratuit, uniformes gratuits, etc.)

c. Un programme de bourses d'études ? Combien de soutien financier serait nécessaire ?

d. Transferts en espèces à vous / votre enfant (conditionnels pour aller à l'école) ?

e. Personnel de sécurité à l'école / sur le chemin de l'école ?

f. Changement de perception des avantages pour l'école ?

g. Avez-vous une proposition ?

(3b) Questionnaire : Version pour les parents avec enfant qui n'est pas en cours de scolarisation

D) L'école et la santé

13) **Pensez-vous que votre enfant apprenais/apprendrait sur la santé à l'école ?**
14) **Pensez-vous que l'école protège des problèmes de santé suivants à court terme (déjà aujourd'hui, cette année) ?**
    a. Santé sexuelle et reproductive, grossesses jeunes, mariages jeunes
    b. Risque d'infection VIH
    c. Comportement risqué : alcool, drogues, ...
    d. Santé mentale, violence et blessures
    e. Accès au système de soins, assurance-maladie
    f. Normes et attitudes
15) **Pensez-vous que l'école protège des problèmes de santé suivants à long terme (dans environ dix ans) ?**
    a. Santé sexuelle et reproductive, grossesses jeunes, mariages jeunes
    b. Risque d'infection VIH
    c. Comportement risqué : alcool, drogues, ...
    d. Santé mentale, violence et blessures
    e. Accès au système de soins, assurance-maladie
    f. Salaires augmentés, opportunités d'emploi améliorés
    g. Normes et attitudes

Suggestions si l'informateur ne sait pas comment l'éducation et la santé peuvent être liées : "Peut-être que le fait d'aller à l'école influence d'une manière que ..."

- Votre enfant passe moins de temps dans la rue -> moins d'occasions de fumer ou de boire de l'alcool ?
- L'école informe votre enfant sur certains sujets afin qu'il ait maintenant un comportement plus sain / moins risqué (nutrition, alcool, tabac, utilisation de préservatifs, ..) ?
- Il trouva des amis ou des modèles qui donnent un bon exemple ?
- Cela lui aide à avoir un meilleur travail / de meilleurs salaires à l'avenir et donc facilite la vie autonome / l'achat de médicaments ?

16) **Dans quelle manière pensez-vous que l'école change les habitudes d'enfants ?** (p. ex. hygiène, comportement risqué,...)
17) **Parlons-nous de vos interactions avec le système de soins**
    a. Savez-vous accéder au système des soins ? (aller au centre de santé / à l'hôpital / à la pharmacie)
    b. Comprenez-vous les informations médicales ? (p. ex. messages de santé publique, ordonnances, information sur la santé sur Internet, ...)
    c. Pensez-vous que ca pourrait faciliter les interactions avec le système de soins que votre enfant aille à l'école ? (p.ex. faciliter la communication avec le personnel de la santé)
    d. A votre enfant vous-a-t-il déjà aidé dans des interactions avec le système de santé ?

(3b) Questionnaire : Version pour les parents avec enfant qui n'est pas en cours de scolarisation

e. Pensez-vous que ca pourrait améliorer votre santé que votre enfant aille à l'école ?

E) Seulement si enfant a abandonné l'école :

E) Effets de débordement

18) **Est-ce que votre enfant partage les avantages qu'il a acquis à l'école ?**
   - **a.** Si oui - avec qui ? Frères et sœurs ? Vous ? Autres ?
   - **b.** Lesquels ? Connaissance ? Les salaires résultant de meilleurs emplois ?

F) Attentes

19) **Pensez-vous que votre enfant pourrait bénéficier d'aller à l'école ?**
   - **a.** Concernant le travail dans le futur ?
   - **b.** Sera-t-il plus heureux / en meilleure santé / ... ?
20) **Vous attendez-vous à ce qu'il prenne soins de vous quand vous sera vieux ?**
   - **a.** *Si oui :* Pensez-vous que la scolarisation pourrait aider à qu'il votre enfant prenne soins de vous ou pensez-vous que cela empêche ?

G) Fin

21) **Y a-t-il quelque chose que je ne vous ai pas demandé et que j'aurais dû vous demander ?**
   - **a.** *Si le répondant dit quelque chose, posez cette question*
22) **Y a-t-il autre chose que vous aimeriez ajouter ?**
23) **Je cherche à rassembler autant d'informations que possible sur l'expérience burkinabè en termes d'accès et de bénéfices perçus pour l'école. Avec qui d'autre me recommanderiez-vous parler ? S'il vous plaît donnez-moi l'orthographe et si possible des informations de contact pour cette personne.**

(4) Questionnaire : Version pour les professeurs

Pour commencer, laissez-moi vous remercier de parler avec moi aujourd'hui. Permettez-moi également de dire que pour les questions que je vais vous poser, il n'y a pas de bonnes ou de mauvaises réponses à mes questions. Je suis ici pour apprendre de vous à cause de vos expériences et de vos points de vue sur la scolarisation au Burkina Faso. Je suis ici pour recueillir des informations auprès de vous afin que d'autres (au Burkina Faso, en Afrique subsaharienne et dans le monde) puissent en apprendre davantage sur vos expériences et qu'ils puissent utiliser ces informations pour réaliser ou modifier d'autres programmes scolaires ou politiques dans des contextes similaires. Tout ce que vous dites sera anonymisé, ce qui signifie que personne ne pourra savoir qui a fourni cette information plus tard.

A) Introduction

1) **Qu'est—ce que vous considérez des points forts / des points faibles du système éducatif au Burkina Faso ?**
   a. A-t-il eu des changements dans les dernières années ? Étaient-elles positives ou négatives ?
   b. A-t-il des différences entre les écoles publiques, privés et religieux ? Lesquelles ?
   c. Vos propositions pour améliorer les écoles / le système éducatif ?
   d. Qu'est-ce que vous considériez comme un système scolaire idéal ?
2) **Qu'est-ce que vous en pensez de l'infrastructure de votre école ?**
   a. Matériel comme cahiers et crayons fournis ? En quantité suffisante ?
   b. Facilités sanitaires (latrines) séparés (homme/femme) ?
   c. A-t-il de l'eau (buvable) ?
   d. A-t-il assez de chaises, tables, ventilateurs, etc. ?
   e. Capacité d'accueil d'élèves ? (A-t-il assez de salles, ...)
   f. Est le bâtiment bon ? (avec fenêtres, ...)
   g. Matériaux pour les étudiants ?
   h. La scolarité d'autres professeurs ?

B) Obstacles

3) **Qu'en est-il des compétences linguistiques des élèves ?**
   a. Quand ils entrent à l'école, ont-ils un niveau de français suffisant pour l'enseignement ?
   b. À partir de quelle année savent-ils bien écrire et lire le francais ?
4) **Où voyez-vous les principaux obstacles pour que les enfants aillent à l'école et restent à l'école ?**
   a. *Comme question générale de départ :* Quelle est la chose avec laquelle la plupart des élèves ont des difficultés concernant aller à l'école ?
   b. Considérez-vous les frais scolaires trop hauts ?
   c. Barrière de la langue ?
   d. Le climat ? Si oui : À cause de quel climat? (chaleur, saison des pluies, ...)
   e. Manque de soutien à la maison ?
   f. Diriez-vous que les élèves sont encouragés à la maison pour aller à l'école ?
   g. Quelles sont les attentes des parents ?

Sondes :

(4) Questionnaire : Version pour les professeurs

1. *Économique*
   - Coûts directs (frais de scolarité secondaire, frais d'appoint non réglementés etc.)
   - Coûts indirects (fournitures, déplacements, coûts d'opportunité, etc.)
2. *Socio-culturelle*
   - Biais de genre : mariages précoces
   - Peer-effets ou d'autres effets de réseau
3. *Psychologique*
   - Besoin perçu, qualité et rendement de la scolarité chez les enfants / parents
   - Redoublement : anxiété
4. *Santé*
   - Santé sexuelle et reproductive : grossesses précoces
   - Santé parentale ; sécurité alimentaire des ménages
5. *Structurel*
   - Distance à l'école secondaire
   - Barrières linguistiques : manque d'infrastructure (p.ex. toilettes séparés pour les filles)
   - Calendrier scolaire inapproprié
6. *Politique et légal*
   - Les lois sur l'éducation obligatoire ; lois sur le travail des enfants
   - Age légal du mariage (17 et dans certains cas 15)
7. *Sécurité*
   - Voyage dangereux à l'école
   - Violence à l'école (pairs, attaques pat des groupes militants)
8. *Géographique*
   - Climat ; saison pluvieuse vs. saison sèche
9. *Historique*
   - Ségrégation

5) **Pendant quelle transition c'est le plus difficile pour les élèves de rester / revenir à l'école ?**
   a. "Préscolaire" à "primaire" (à l'âge de 6 ans environ)
   b. "Primaire" à "post-primaire" (à l'âge de 12 ans environ)
   c. "Post-primaire" à "lycée" / continuer après "post-primaire" (à l'âge de 16 ans environ)
6) **Pourquoi c'est en ce moment là que c'est si difficile ?**

C) Interventions possibles

7) **Qu'est-ce qui rendrait plus facile pour les élèves d'aller à l'école / pour leurs parents de les envoyer à l'école ?**

(4) Questionnaire : Version pour les professeurs

a. Une moindre distance à l'école ?
b. Des frais moins élevés pour l'école ? Abaisser les dépenses supplémentaires ? (Par exemple par transport gratuit, uniformes gratuits, etc.)
c. Un programme de bourses d'études ? Combien de soutien financier serait nécessaire ?
d. Transferts en espèces aux élèves / leurs parents (conditionnels pour aller à l'école) ?
e. Personnel de sécurité à l'école / sur le chemin de l'école ?
f. Changement de perception des avantages pour l'école ?
g. Avez-vous une proposition ?

D) L'école et la santé

8) **Pensez-vous que l'école protège des problèmes de santé suivants à court terme (déjà aujourd'hui, cette année) ?**
   a. Santé sexuelle et reproductive, grossesses jeunes, mariages jeunes
   b. Risque d'infection VIH
   c. Comportement risqué : alcool, drogues, ...
   d. Santé mentale, violence et blessures
   e. Accès au système de soins, assurance-maladie
   f. Normes et attitudes
9) **Pensez-vous que l'école protège des problèmes de santé suivants à long terme (dans environ dix ans) ?**
   a. Santé sexuelle et reproductive, grossesses jeunes, mariages jeunes
   b. Risque d'infection VIH
   c. Comportement risqué : alcool, drogues, ...
   d. Santé mentale, violence et blessures
   e. Accès au système de soins, assurance-maladie
   f. Salaires augmentés, opportunités d'emploi améliorés
   g. Normes et attitudes

Suggestions si l'informateur ne sait pas comment l'éducation et la santé peuvent être liées : "Peut-être que le fait d'aller à l'école influence d'une manière que ..."

- L'élève passe moins de temps dans la rue -> moins d'occasions de fumer ou de boire de l'alcool ?
- L'école informe sur certains sujets afin que l'élève maintenant un comportement plus sain / moins risqué (nutrition, alcool, tabac, utilisation de préservatifs, ..) ?
- Il trouva des amis ou des modèles qui donnent un bon exemple ?
- Cela lui aide à avoir un meilleur travail / de meilleurs salaires à l'avenir et donc facilite la vie autonome / l'achat de médicaments ?

10) **La santé dans le curriculum :**
   a. Traitez-vous des sujets de santé (IST, comportement à risque, utilisation de préservatifs ...) à l'école?
   b. Comment réagissent les élèves à cela ?

c. Pensez-vous que cela a une influence positive sur leur santé ? (interactions plus facile avec le système de soins, moins comportement risqué) Avez-vous des exemples ?

E) Effets de débordement

11) **Est-ce que les élèves partagent les avantages acquis à l'école avec les gens autour d'eux ?**
    a. Si oui - avec qui ? Frères et sœurs ? Vous ? Autres ?
    b. Lesquels ? Connaissance ? Les salaires résultant de meilleurs emplois ?

F) Assiduité des élèves

12) **Qu'en est-il de la présence des élèves à l'école ?**
    a. La taille des classes est-elle stable ?
    b. Combien d'étudiants ne sont régulièrement pas à l'école ?
    c. Est-ce que les étudiants abandonnent fréquemment ?
    d. Le suivi des élèves au fil du temps est-il possible avec la plupart des élèves ou avec de petit nombre d'élèves ?
    e. Y a-t-il des listes de présence / «pénalités» pour ne pas être présent?
13) **Quelles sont les raisons pour lesquelles les élèves ne sont pas présents ?**
    a. Quelle est la principale raison pour laquelle les élèves manquent la classe ? (maladie, obligation de travail à la maison, ...)
    --> Voir les sondes de la question 4
    b. Quelles sont les raisons de l'abandon ?
    --> Voir les sondes de la question 4
    c. Voyez-vous des associations fréquentes ?
    d. Connaissez-vous les familles des élèves ? Comment décririez-vous la relation avec eux ?
    e. Que faites-vous pour
        i. Empêcher l'abandon de l'école par l'élève ?
        ii. Améliorer la fréquentation scolaire ?
14) **Y a-t-il une différence entre les filles et les garçons ?**
    a. Est-il plus facile d'aller à l'école pour un sexe que pour l'autre ?
    b. Pourquoi ?

G) Fin

15) **Y a-t-il quelque chose que je ne vous ai pas demandé et que j'aurais dû vous demander ?**
    a. *Si le répondant dit quelque chose, posez cette question*
16) **Y a-t-il autre chose que vous aimeriez ajouter ?**
17) **Je cherche à rassembler autant d'informations que possible sur l'expérience burkinabè en termes d'accès et de bénéfices perçus pour l'école. Avec qui d'autre me recommanderiez-vous parler ? S'il vous plaît donnez-moi l'orthographe et si possible des informations de contact pour cette personne.**

(4) Questionnaire : Version pour les professeurs

(5) Questionnaire : Version pour un représentant de l’Association des Parents

Pour commencer, laissez-moi vous remercier de parler avec moi aujourd'hui. Permettez-moi également de dire que pour les questions que je vais vous poser, il n'y a pas de bonnes ou de mauvaises réponses à mes questions. Je suis ici pour apprendre de vous à cause de vos expériences et de vos points de vue sur la scolarisation au Burkina Faso. Je suis ici pour recueillir des informations auprès de vous afin que d'autres (au Burkina Faso, en Afrique subsaharienne et dans le monde) puissent en apprendre davantage sur vos expériences et qu'ils puissent utiliser ces informations pour réaliser ou modifier d'autres programmes scolaires ou politiques dans des contextes similaires. Tout ce que vous dites sera anonymisé, ce qui signifie que personne ne pourra savoir qui a fourni cette information plus tard.

Je vous prie de prendre le rôle de représenter tous les parents de votre école et village, de tenir en compte la totalité des parents, leurs difficultés et possibilités.

A) Introduction

1) **Qu’est—ce que vous considérez des points forts / des points faibles du système éducatif au Burkina Faso ?**
    a. A-t-il eu des changements dans les dernières années ? Étaient-elles positives ou négatives ?
    b. A-t-il des différences entre les écoles publiques, privés et religieux ? Lesquelles ?
    c. Vos propositions pour améliorer les écoles / le système éducatif ?
    d. Qu’est-ce que vous considériez comme un système scolaire idéal ?

B) Obstacles

2) **Où voyez-vous les principaux obstacles pour les parents d’envoyer les enfants à l'école et qu’ils puissent rester à l'école ?**
    a. *Comme question générale de départ :* Quelle est la chose avec laquelle la plupart des parents ont des difficultés concernant la scolarisation de leurs enfants ?
    b. Considérez-vous les frais scolaires trop hauts ?
    c. Barrière de la langue ? (Langue locale à la maison compète avec l’apprentissage du français à l’école)
    d. Le climat ? Si oui : À cause de quel climat ? (Chaleur, saison des pluies, ...)
    e. Manque de conscience sur les bénéfices de l’école ?
    f. Diriez-vous que les enfants sont encouragés à la maison pour aller à l'école ?
    g. Quelles sont les attentes des parents concernant la scolarisation de leurs enfants ?
3) **Lesquelles sont les dépenses les plus difficiles de payer pour les parents ?** *(Le participant doit choisir 2 dépenses)*
4) **Si vous pensez aux parents qui n’envoient pas leurs enfants à l’école : C’est pourquoi ?**
    après réponse demander de spécifier :
    a. Plutôt par rapport à des raisons extérieures (p.ex. manque de moyens financiers) ?
    où
    b. Plutôt parce que les parents (et leurs enfants) ne voient pas de nécessité / pas d’avantages de l’école ?

(5) Questionnaire : Version pour un représentant de l'Association des Parents

Sondes :

1. *Économique*
    - Coûts directs (frais de scolarité secondaire, frais d'appoint non réglementés etc.)
    - Coûts indirects (fournitures, déplacements, coûts d'opportunité, etc.)
2. *Socio-culturelle*
    - Biais de genre : mariages précoces
    - Peer-effets ou d'autres effets de réseau
3. *Psychologique*
    - Besoin perçu, qualité et rendement de la scolarité chez les enfants / parents
    - Redoublement : anxiété
4. *Santé*
    - Santé sexuelle et reproductive : grossesses précoces
    - Santé parentale ; sécurité alimentaire des ménages
5. *Structurel*
    - Distance à l'école secondaire
    - Barrières linguistiques : manque d'infrastructure (p.ex. toilettes séparés pour les filles)
    - Calendrier scolaire inapproprié
6. *Politique et légal*
    - Les lois sur l'éducation obligatoire ; lois sur le travail des enfants
    - Age légal du mariage (17 et dans certains cas 15)
7. *Sécurité*
    - Voyage dangereux à l'école
    - Violence à l'école (pairs, attaques pat des groupes militants)
8. *Géographique*
    - Climat ; saison pluvieuse vs. saison sèche
9. *Historique*
    - Ségrégation

5) **Pendant quelle transition c'est le plus difficile pour les élèves de rester / revenir à l'école ?**
    a. L'entrée au primaire
    b. Du primaire (CM2) au 6ème
    c. Du 3ème au 2nde (collège au lycée)
6) **Pourquoi c'est en ce moment-là que c'est si difficile ?**

(5) Questionnaire : Version pour un représentant de l'Association des Parents

C) Mouvement des parents

7) **I-a-t-il déjà des mouvements/ initiatives du côté des parents ?**
8) **Qu'est-ce que sont les activités de l'association des parents ? Qu'est-ce que vous faites pour le fonctionnement de l'école ?**
    a. En général, au niveau des élèves et des professeurs, pour améliorer la fréquentation de l'école
    b. Pour aider aux autres parents par rapport aux difficultés retrouvés dans le cadre de la scolarisation)

D) Qualité de la scolarisation

9) **Qu'est-ce que vous en pensez de la qualité de la scolarisation ?**
    a. Par rapport à la qualité des professeurs (bien formés/mal formés ?)
    b. Quels autres facteurs diminuent la qualité de l'école ?

E) Interventions possibles

10) **Qu'est-ce qui rendrait plus facile pour les élèves d'aller à l'école / pour leurs parents de les envoyer à l'école ? Est-ce que vous avez une proposition ?**
    Si la personne n'a pos des idées on peut proposer :
    a. Une moindre distance à l'école ?
    b. Des frais moins élevés pour l'école ? Abaisser les dépenses supplémentaires ? (Par exemple par transport gratuit, uniformes gratuits, etc.)
    c. Un programme de bourses d'études ? Combien de soutien financier serait nécessaire ?
    d. Transferts en espèces aux élèves / leurs parents (conditionnels pour aller à l'école) ?
    e. Personnel de sécurité à l'école / sur le chemin de l'école ?
    f. Changement de perception des avantages pour l'école ?
11) **C'est quoi l'intervention la plus puissante / la plus utile pour faciliter aux parents d'envoyer leurs enfants à l'école ? / Pour améliorer la fréquentation de l'école ?**
    Attendre d'abord l'idée du parent. Après on veut couvrir les sujets suivants : « C'est le plus utile de ... » :
    a. Amener de l'électricité
        i. A l'école ?
        ii. Aux ménages ?
    b. Amener de l'eau (buvable/potable)
    c. Offrir le déjeuner à l'école
        i. Doit ça être gratuit ?
    d. Disposer des moyens de transport pour aller à l'école ?
        i. Lesquelles ? : Vélos, bus, ...
    e. Campagne de sensibilisation sur les bénéfices de la scolarité ?

(5) Questionnaire : Version pour un représentant de l'Association des Parents

F) L'école et la santé

12) **Pensez-vous que les gens voient un rapport entre l'éducation et la santé ?** (Que l'éducation apporte à une meilleure santé) A long terme / à court terme
13) **Dans quelle manière ?**
    Si le participant n'a pas d'idées aider avec :
    a. Santé sexuelle et reproductive, grossesses jeunes, mariages jeunes
    b. Risque d'infection VIH
    c. Comportement risqué : alcool, drogues, ...
    d. Santé mentale, violence et blessures
    e. Accès au système de soins, assurance-maladie
    f. Salaires augmentés, opportunités d'emploi améliorés
    g. Normes et attitudes
14) **Est-ce que l'éducation des enfants facilite l'accès aux soins de son entourage ? Comment ?**

G) Effets de débordement

15) **Est-ce que vous les élèves partagent les avantages acquis à l'école avec les gens autour d'eux ?**
    a. Si oui - avec qui ? Frères et sœurs ? Vous ? Autres ?
    b. Lesquels ? Connaissance ? Les salaires résultant de meilleurs emplois ?
16) **Comment est-ce que ça peut apporter des bénéfices au niveau de famille / au niveau du village ?**

H) Fin

17) **Y a-t-il autre chose que vous aimeriez ajouter ?**

Perceptions relatives à la santé et l'école secondaire : une étude utilisant des méthodes mixtes au Burkina Faso

(6) Questionnaire : Version pour les Tuteurs

Pour commencer, laissez-moi vous remercier de parler avec moi aujourd'hui. Permettez-moi également de dire que pour les questions que je vais vous poser, il n'y a pas de bonnes ou de mauvaises réponses à mes questions. Je suis ici pour apprendre de vous à cause de vos expériences et de vos points de vue sur la scolarisation au Burkina Faso. Je suis ici pour recueillir des informations auprès de vous afin que d'autres (au Burkina Faso, en Afrique subsaharienne et dans le monde) puissent en apprendre davantage sur vos expériences et qu'ils puissent utiliser ces informations pour réaliser ou modifier d'autres programmes scolaires ou politiques dans des contextes similaires. Tout ce que vous dites sera anonymisé, ce qui signifie que personne ne pourra savoir qui a fourni cette information plus tard.

1) Pourquoi est l'élève chez vous ?

2) Comment ça se passe avec l'élève ?

3) **Quels problèmes se posent ?**
   a) Au niveau financier
   b) Au niveau de la vie commune

4) Quelle est votre motivation pour accueillir l'élève ?

5) Qu'est-ce qu'il faut fournir pour la scolarisation de l'élève et qui paye quoi ?

6) **Est-ce que l'élève doit aider avec des travaux ? Lesquelles ?**
   p.ex. : obligations dans le ménage, travailler à côté de l'école hors du ménage, travailler avec le tuteur dans ce qu'il travail lui

7) **Est-ce que l'élève doit compenser pour / payer les dépenses prise en charge par le tuteur d'une autre manière ?** (P.ex. comportement risqué ou des faveurs au tuteur)

8) **Selon vous : Quels sont les difficultés retrouvées par l'élève ?**
   a) Pour aller à l'école
   b) Pour réussir à l'école
   c) Au niveau de la vie quotidienne dans votre ménage et loin de sa famille
   d) Concernant les allers – retours chez sa famille

9) **Selon vous : Pourquoi est-ce que les parents de l'élève font cet effort pour l'envoyer à l'école ?**
   Ils voient quels avantages ? Ils ont quelles attentes ?

10) **Quelle relation à l'élève avec la scolarisation ?**
   a) Veut-il aller à l'école lui-même ? Est-ce qu'il aime bien fréquenter ?

(6) Questionnaire : Version pour les Tuteurs

b) Assiduité : Fréquente-il régulièrement ?

c) *Si « non » :* Qu'est-ce que lui empêchent ? Ou est-ce qu'il n'a seulement pas envie ?

11) Quels sont les avantages pour l'élève d'être chez vous / d'être dans cette situation ?

12) Quels sont les avantages pour vous de recevoir l'élève ?

13) Profitez-vous ou l'entourage de l'élève des connaissances de l'élève ?

a) En général

b) Par rapport à l'accès au système de soins

c) Partage-t-il ces connaissances ? Avec qui ?

14) **Qu'est-ce qu'on pourrait faire pour lui faciliter d'aller à l'école ?**

15) **Qu'est-ce qu'on pourrait faire pour vous de faciliter qu'il aille à l'école ?**

16) **Aimeriez-vous rajouter quelque chose ?**

(7) Questionnaire : Version pour les directeurs des collèges et des lycées

Pour commencer, laissez-moi vous remercier de parler avec moi aujourd'hui. Permettez-moi également de dire que pour les questions que je vais vous poser, il n'y a pas de bonnes ou de mauvaises réponses à mes questions. Je suis ici pour apprendre de vous à cause de vos expériences et de vos points de vue sur la scolarisation au Burkina Faso. Je suis ici pour recueillir des informations auprès de vous afin que d'autres (au Burkina Faso, en Afrique subsaharienne et dans le monde) puissent en apprendre davantage sur vos expériences et qu'ils puissent utiliser ces informations pour réaliser ou modifier d'autres programmes scolaires ou politiques dans des contextes similaires. Tout ce que vous dites sera anonymisé, ce qui signifie que personne ne pourra savoir qui a fourni cette information plus tard.

A) Introduction

1) **Qu'est—ce que vous considérez des points forts / des points faibles du système éducatif au Burkina Faso ?**
    a. A-t-il eu des changements dans les dernières années ? Étaient-elles positives ou négatives ?
    b. A-t-il des différences entre les écoles publiques, privés et religieux ? Lesquelles ?
2) **Quelles sont les difficultés que vous rencontrez tant que directeur de collège ?**
3) **Quelles sont les difficultés les plus importantes ?**

B) Le système éducatif, l'état et la politique

4) **Pouvez-vous me raconter sur les acteurs impliqués dans le système éducatif et la scolarisation ?** Ce sont lesquels ? Qu'est-ce qu'ils font ? Qu'est-ce qu'ils sont leurs intérêts ?
5) **Qu'est-ce que vous pouvez me raconter sur les programmes de l'état concernant l'éducation ?** VOUS LES CONSIDÉREZ UTILES OU NON ? LESQUELS ? POURQUOI ?
6) **TANT QU'AUX MOYENS POUR L'ÉDUCATION : D'OU EST-CE QU'ILS VIENNENT ?** (Impôts, des organisations de la communauté mondial p.ex. FMI)
7) **Qu'est-ce que vous feriez tant que l'état pour améliorer la situation des écoles / la qualité d'enseignement / la fréquentation de l'école ?**
    a. Comment-devrait ça être réalisé ? (P.ex. diminuer un autre budget de l'état pour augmenter le budget pour l'éducation, augmenter les impôts, fonds de l'extérieur)
8) **Savez-vous de mouvements qui s'engagent pour l'amélioration du système éducatif ou engagez-vous vous-même ?**

C) Obstacles

9) **Selon vous : Quels sont les obstacles les plus fréquents pour les élèves de fréquenter l'école / pour les parents d'envoyer leurs enfants à l'école ?**
10) **Quels sont les empêchements les plus importants pour les parents/élèves pour l'entrée au parcours scolaire ?** (Ça veut dire pour entrer au primaire)
11) **Dans quel moment du parcours éducatif d'un enfant c'est le plus difficile rester à l'école ?** (Ça veut dire la transition du primaire (CM2) au secondaire (6ème) ou la transition du collège au lycée (3ème au 2nde))
12) **Pourquoi ?**

(7) Questionnaire : Version pour les directeurs des collèges et des lycées

D) Interventions

13) **Donnez-moi deux propositions qui seraient les plus puissantes pour améliorer la situation à l'école.**

Si la personne n'a pos des idées on peut proposer ou on veut donner plus d'idées :

a. Amener de l'électricité
    i. A l'école ?
    ii. Aux ménages ?
b. Amener de l'eau (buvable/potable)
c. Améliorations dans le bâtiment :
    i. Fenêtres ou similaires
    ii. Ventilateurs ou similaires
    iii. Plus de tables / chaises ou similaires
d. Meilleure disponibilité de matériel scolaire
    i. P.ex. imprimantes à l'école ou autre qui facilite la disponibilité de photocopies
    ii. Plus de matériel de cours, matériel de cours plus récents (pour le professeur et les élèves)

14) **Donnez-moi deux propositions qui seraient les plus puissantes pour faciliter la fréquentation de l'école.**

Si la personne n'a pos des idées on peut proposer ou on veut donner plus d'idées :

a. Offrir le déjeuner à l'école
    i. Doit ça être gratuit ?
b. Disposer des moyens de transport pour aller à l'école ?
    i. Lesquelles ? : Vélos, bus, ...
c. Diminution / abolition des frais scolaires
d. Diminution / abolition des dépenses supplémentaires
e. Transfert en espèces à l'élève ou plutôt au parent ? Combien de l'argent par mois serait nécessaire ?
f. Campagne de sensibilisation sur les bénéfices de la scolarité ?

E) L'école et la santé

15) **Quel est le rapport entre l'éducation et la santé ?**
16) **Comment est-ce qu'on peut promouvoir celui-là au pour améliorer les résultats de santé ?**
    a. A court terme
    b. A long terme

F) L'avenir

17) **Comment-est-ce que vous voyez l'avenir du système éducatif au Burkina ?**
18) **Qu'est-ce que vous souhaiteriez pour le système éducatif au Burkina ?**

D) Fin

19) **Y a-t-il autre chose que vous aimeriez ajouter ?**

(7b) Questionnaire : Version pour le syndicat des professeurs

Pour commencer, laissez-moi vous remercier de parler avec moi aujourd'hui. Permettez-moi également de dire que pour les questions que je vais vous poser, il n'y a pas de bonnes ou de mauvaises réponses à mes questions. Je suis ici pour apprendre de vous à cause de vos expériences et de vos points de vue sur la scolarisation au Burkina Faso. Je suis ici pour recueillir des informations auprès de vous afin que d'autres (au Burkina Faso, en Afrique subsaharienne et dans le monde) puissent en apprendre davantage sur vos expériences et qu'ils puissent utiliser ces informations pour réaliser ou modifier d'autres programmes scolaires ou politiques dans des contextes similaires. Tout ce que vous dites sera anonymisé, ce qui signifie que personne ne pourra savoir qui a fourni cette information plus tard.

Je vous prie de répondre comme représentant des enseignants et tenir en compte les difficultés de la totalité des enseignants et les priorités des celles.

A) Introduction

1) **Qu'est—ce que vous considérez des points forts / des points faibles du système éducatif au Burkina Faso ?**
    a. A-t-il eu des changements dans les dernières années ? Étaient-elles positives ou négatives ?
    b. A-t-il des différences entre les écoles publiques, privés et religieux ? Lesquelles ?
2) **Quelles sont les difficultés que vous rencontrez tant que enseignants ?**
3) **Quelles sont les difficultés les plus importantes ?**

B) Le système éducatif, l'état et la politique

4) **Pouvez-vous me raconter sur les acteurs impliqués dans le système éducatif et la scolarisation ?** Ce sont lesquels ? Qu'est-ce qu'ils font ? Qu'est-ce qu'ils sont leurs intérêts ?
5) **Qu'est-ce que vous pouvez me raconter sur les programmes de l'état concernant l'éducation ?** VOUS LES CONSIDÉREZ UTILES OU NON ? LESQUELS ? POURQUOI ?
6) **TANT QU'AUX MOYENS POUR L'ÉDUCATION : D'OU EST-CE QU'ILS VIENNENT ?** (Impôts, des organisations de la communauté mondial p.ex. FMI)
7) **Qu'est-ce que vous feriez tant que l'état pour améliorer la situation des écoles / la qualité d'enseignement / la fréquentation de l'école ?**
    a. Comment-devrait ça être réalisé ? (P.ex. diminuer un autre budget de l'état pour augmenter le budget pour l'éducation, augmenter les impôts, fonds de l'extérieur)
8) **Savez-vous de mouvements qui s'engagent pour l'amélioration du système éducatif ou engagez-vous vous-même ?**
9) **Comment est le syndicat organisé ?** Combien de personnes est-ce qu'il y a, ... ?
10) **Quelles sont les activités du syndicat ?**

C) Obstacles

11) **Selon vous : Quels sont les obstacles les plus fréquents pour les élèves de fréquenter l'école / pour les parents d'envoyer leurs enfants à l'école ?**
12) **Quels sont les empêchements les plus importants pour les parents/élèves pour l'entrée au parcours scolaire ?** (Ça veut dire pour entrer au primaire)

(7b) Questionnaire : Version pour le syndicat des professeurs

13) **Dans quel moment du parcours éducatif d'un enfant c'est le plus difficile rester à l'école ?** (Ça veut dire la transition du primaire (CM2) au secondaire (6ème) ou la transition du collège au lycée (3ème au 2nde))
14) **Pourquoi ?**

D) Interventions

15) **Donnez-moi deux propositions qui seraient les plus puissantes pour améliorer la situation à l'école.**
Si la personne n'a pos des idées on peut proposer ou on veut donner plus d'idées :
    a. Amener de l'électricité
        i. A l'école ?
        ii. Aux ménages ?
    b. Amener de l'eau (buvable/potable)
    c. Améliorations dans le bâtiment :
        i. Fenêtres ou similaires
        ii. Ventilateurs ou similaires
        iii. Plus de tables / chaises ou similaires
    d. Meilleure disponibilité de matériel scolaire
        i. P.ex. imprimantes à l'école ou autre qui facilite la disponibilité de photocopies
        ii. Plus de matériel de cours, matériel de cours plus récents (pour le professeur et les élèves)

16) **Donnez-moi deux propositions qui seraient les plus puissantes pour faciliter la fréquentation de l'école.**
Si la personne n'a pos des idées on peut proposer ou on veut donner plus d'idées :
    a. Offrir le déjeuner à l'école
        i. Doit ça être gratuit ?
    b. Disposer des moyens de transport pour aller à l'école ?
        i. Lesquelles ? : Vélos, bus, ...
    c. Diminution / abolition des frais scolaires
    d. Diminution / abolition des dépenses supplémentaires
    e. Transfert en espèces à l'élève ou plutôt au parent ? Combien de l'argent par mois serait nécessaire ?
    f. Campagne de sensibilisation sur les bénéfices de la scolarité ?

E) L'école et la santé

17) **Quel est le rapport entre l'éducation et la santé ?**
18) **Comment est-ce qu'on peut promouvoir celui-là au pour améliorer les résultats de santé ?**
    a. A court terme
    b. A long terme

F) L'avenir

19) **Comment-est-ce que vous voyez l'avenir du système éducatif au Burkina ?**

(7b) Questionnaire : Version pour le syndicat des professeurs

20) **Qu’est-ce que vous souhaiteriez pour le système éducatif au Burkina ?**

D) <u>Fin</u>

21) **Y a-t-il autre chose que vous aimeriez ajouter ?**

(8) Questionnaire : Version pour les CSPS

Pour commencer, laissez-moi vous remercier de parler avec moi aujourd'hui. Permettez-moi également de dire que pour les questions que je vais vous poser, il n'y a pas de bonnes ou de mauvaises réponses à mes questions. Je suis ici pour apprendre de vous à cause de vos expériences et de vos points de vue sur la scolarisation au Burkina Faso. Je suis ici pour recueillir des informations auprès de vous afin que d'autres (au Burkina Faso, en Afrique subsaharienne et dans le monde) puissent en apprendre davantage sur vos expériences et qu'ils puissent utiliser ces informations pour réaliser ou modifier d'autres programmes scolaires ou politiques dans des contextes similaires. Tout ce que vous dites sera anonymisé, ce qui signifie que personne ne pourra savoir qui a fourni cette information plus tard.

1) Voyez-vous un rapport entre l’éducation d’une personne et sa santé ? Lequel/Lesquels ? Des bénéfices ?
   a. A court terme
   b. A long terme
   c. C’est du à quoi ? / Pourquoi est-ce qu’il y a un rapport ?

2) **Si vous regardez les gens qui viennent au CSPS : Comment décrieriez-vous leur niveau scolaire ?**
3) **Diriez vous que les gens qui n’étaient pas à l’école ou les gens qui étaient à l’école moins longtemps fréquentent moins le CSPS que ceux avec un niveau scolaire plus élevé ?**
   a. Pourquoi ? Quelles sont les raisons ?
4) **Avec les patients : Est-ce que vous vous trouvez confronté à des difficultés à cause de leur niveau scolaire ?**
   a. Lesquels ?
   b. Comment est-ce que vous affrontez ces difficultés ?
   c. Pouvez-vous me raconter une expérience comme ça ?

5) **Selon vous : Comment facilite l’éducation l’accès au système de soins ?**
   a. Est-ce qu’elle facilite seulement l’accès au système de soins pour la personne formée ? Ou aussi pour son entourage ?
   b. Pour qui ?
   c. Compréhension d’ordonnances, messages de santé publique
6) **Voyez-vous des enfants (élèves) qui accompagnent p.ex. leurs parents (ou des amis analphabètes ou ...) ?**
   a. Est-ce que ça arrive souvent ?
   b. Pourquoi est-ce qu’ils les accompagnent ?
   c. Comment est-ce qu’ils les aident ?
   d. Est-ce que ça vous facilite le travail à vous ?

(8) Questionnaire : Version pour les CSPS

7) **En quoi diffèrent les enfants qui vont à l'école comparé à ceux qui ne fréquentent pas par rapport à leurs connaissances dans le domaine de la santé ?**

8) **Pensez-vous que l'école protège des problèmes de santé suivants à court terme (déjà aujourd'hui, cette année) ?**
    a. Santé sexuelle et reproductive, grossesses jeunes, mariages jeunes
    b. Risque d'infection VIH
    c. Comportement risqué : alcool, drogues, ...
    d. Santé mentale, violence et blessures
    e. Accès au système de soins, assurance-maladie
    f. Normes et attitudes
9) **Pensez-vous que l'école protège des problèmes de santé suivants à long terme (dans environ dix ans) ?**
    a. Santé sexuelle et reproductive, grossesses jeunes, mariages jeunes
    b. Risque d'infection VIH
    c. Comportement risqué : alcool, drogues, ...
    d. Santé mentale, violence et blessures
    e. Accès au système de soins, assurance-maladie
    f. Salaires augmentés, opportunités d'emploi améliorés
    g. Normes et attitudes

10) **Pensez-vous que les personnes formées ont des habitudes moins malsaines que ceux avec un niveau scolaire plus bas ?** (P.ex. sport, alimentation, tabac, alcool, autres drogues)
11) **Est-ce que le cas de problème de santé varie selon le niveau d'instruction ?** (Observez-vous p.ex. plus d'accidents de moto chez les personnes d'un niveau d'instruction plus/moins élevé ?)

12) **Y a-t-il autre chose que vous aimeriez ajouter ?**

(9) Questionnaire : Version pour le DEPNA

Pour commencer, laissez-moi vous remercier de parler avec moi aujourd'hui. Permettez-moi également de dire que pour les questions que je vais vous poser, il n'y a pas de bonnes ou de mauvaises réponses à mes questions. Je suis ici pour apprendre de vous à cause de vos expériences et de vos points de vue sur la scolarisation au Burkina Faso. Je suis ici pour recueillir des informations auprès de vous afin que d'autres (au Burkina Faso, en Afrique subsaharienne et dans le monde) puissent en apprendre davantage sur vos expériences et qu'ils puissent utiliser ces informations pour réaliser ou modifier d'autres programmes scolaires ou politiques dans des contextes similaires. Tout ce que vous dites sera anonymisé, ce qui signifie que personne ne pourra savoir qui a fourni cette information plus tard.

A) Introduction

1) **Qu'est—ce que vous considérez des points forts / des points faibles du système éducatif au Burkina Faso ?**
    a. A-t-il eu des changements dans les dernières années ? Étaient-elles positives ou négatives ?
    b. A-t-il des différences entre les écoles publiques, privés et religieux ? Lesquelles ?
2) **Quelles sont les difficultés les plus importantes ? Où voyez-vous des défauts dans le fonctionnement du système et du DEPNA ?**

B) Le système éducatif, l'état et la politique

3) **Pouvez-vous me raconter sur les acteurs impliqués dans le système éducatif et la scolarisation ?** Ce sont lesquels ? Qu'est-ce qu'ils font ? Qu'est-ce qu'ils sont leurs intérêts ?
4) **Qu'est-ce que vous pouvez me raconter sur les programmes de l'état concernant l'éducation ?** Vous les considérez utiles ou non ? Pourquoi ?
5) **Tant qu'aux moyens pour l'éducation : Quelles sont les sources du budget pour l'éducation ?** (Impôts, des organisations de la communauté mondial p.ex. FMI)
6) **Qu'est-ce que le DEPNA fait pour améliorer la situation des écoles / la qualité d'enseignement / la fréquentation de l'école ?**
7) **Y-a-t-il des programmes prévus ?**

C) Obstacles

8) **Selon vous : Quels sont les obstacles les plus fréquents pour les élèves de fréquenter l'école / pour les parents d'envoyer leurs enfants à l'école ?**
9) **Quels sont les empêchements les plus importants pour les parents/élèves pour l'entrée au parcours scolaire ?** (Ça veut dire pour entrer au primaire)
10) **Dans quel moment du parcours éducatif d'un enfant c'est le plus difficile rester à l'école ?** (Ça veut dire la transition du primaire (CM2) au secondaire (6ème) ou la transition du collège au lycée (3ème au 2nde))
11) **Pourquoi ?**
12) **Pensez-vous que les cycles (préscolaire, primaire, secondaire 1 et 2) représentent des barrières virtuelles, voire réelles ?**

(9) Questionnaire : Version pour le DEPNA

D) Interventions

13) **Donnez-moi deux propositions qui seraient les plus puissantes pour améliorer la situation pour les élèves et les professeurs dans les écoles.**
Si la personne n'a pos des idées on peut proposer ou on veut donner plus d'idées :
   a. Amener de l'électricité
      i. A l'école ?
      ii. Aux ménages ?
   b. Amener de l'eau (buvable/potable)
   c. Améliorations dans le bâtiment :
      i. Fenêtres ou similaires
      ii. Ventilateurs ou similaires
      iii. Plus de tables / chaises ou similaires
   d. Meilleure disponibilité de matériel scolaire
      i. P.ex. imprimantes à l'école ou autre qui facilite la disponibilité de photocopies
      ii. Plus de matériel de cours, matériel de cours plus récents (pour le professeur et les élèves)

14) **Donnez-moi deux propositions qui seraient les plus puissantes pour faciliter la fréquentation de l'école pour les élèves/enfants.**
Si la personne n'a pos des idées on peut proposer ou on veut donner plus d'idées :
   a. Offrir le déjeuner à l'école
      i. Doit ça être gratuit ?
   b. Disposer des moyens de transport pour aller à l'école ?
      i. Lesquelles ? : Vélos, bus, ...
   c. Diminution / abolition des frais scolaires
   d. Diminution / abolition des dépenses supplémentaires
   e. Transfert en espèces à l'élève ou plutôt au parent ? Combien de l'argent par mois serait nécessaire ?
   f. Campagne de sensibilisation sur les bénéfices de la scolarité ?

E) L'école et la santé

15) **Quel rôle joue la santé dans le système éducatif ?**
16) **À quel niveau est la santé tenue en compte ?**
(P.ex. dans curriculum des élèves, comme matière, ...)
17) **Voyez-vous des bénéfices de l'éducation par rapport à la santé ? Comment est-ce que le DEPNA encourage ça ?**

D) Fin

18) **Y a-t-il autre chose que vous aimeriez ajouter ?**
