# Supplementary material for: “It’s the poverty”—Stakeholder perspectives on barriers to secondary education in rural Burkina Faso
Source: PLoS One. 2022 Nov 17;17(11):e0277822. doi: 10.1371/journal.pone.0277822 (PMC9671424; doi:10.1371/journal.pone.0277822)
Supplement: Attachment — Submitted filename: PONE-D-21-34581_comments.pdf [file pone.0277822.s004.pdf]

“Girls were seen as lost investments” – stakeholder perspectives on barriers to secondary education in rural Burkina Faso

May 21, 2022

1 Comments and Suggestions

1.1 Title

- The title “Girls were seen as lost investments” is a compelling one, but gives the impression that the paper as a whole will be about barriers to secondary schooling for (primarily) girls. The authors may want to consider using a different quote for their title.

1.2 Introduction

Pg 4: Describe upfront who counts as a stakeholder in this context.

1.3 Materials and Methods

Pg 5: Under “Study Area”, for the statements about the predominant occupations of residents, ethnic groups, and languages in the Nouna HDSS, are there any citations that you can provide?

Pg 6: Though the authors address in the “Study Limitations” section that there may be bias in their data due to the small sample size, more can be done to address possible sources of bias. For example, providing more detail about the total number of participants invited to interview by participant type (in-school adolescent, out-of-school adolescent, parent, teacher, etc. by gender and other sociodemographic characteristics, if available) and the total number of rejections broken down by those same characteristics would be helpful to understand where bias may lie.

- The sentence in Page 7 under “Study sample” that begins with “Respondents were randomly selected...” would be better placed under the “Study Design” subsection.
- More detail needs to be provided about the second stage sampling – e.g. were there possible sources of bias in the selection of the second stage respondents, given that local study team members were the ones who guided the identification of possible participants?
- Describe the pilot study first, prior to any description about the first stage sampling.

Pg 7:
- Under "Interviews", were the local experts hired and managed directly by the study team, or were they associated with a survey firm of some kind?
- There is a redundant citation under "Study Sample" ([30]).

Pg 8: Provide a formal definition of "resilience" and define what "maladaptive coping strategies" are in this context.

1.4 Results

General comments: First, it's unclear where many of the accounts from - students, out of school adolescents, parents, teachers, or others. This is important for some of the views on barriers to education (e.g. the accounts of students being envious of out-of-school peers in the labor force). If the descriptions came from multiple types of stakeholders, it may benefit the reader to be more explicit.

Second, a clearer distinction should be made about whether some barriers primarily affect boys or affect boys and girls more-or-less equally. For example, the account about the salience of the benefits of not being a student by observing the relative wealth of out-of-school peers seems like a barrier that affects mostly boys, since girls are expected to be homemakers according to the interviews. The language used when describing this observation is gender-neutral, even though the barrier is not.

Lastly, there is an excessive use of the word "would" in much of this section. Many of the accounts are of events or observations that have already happened, but this passive language makes it sound like some of these events have not happened yet. The language needs to be amended.

Pg 12: There is a typo: "–;" should be changed to "–".

Pg 13:
- Does the statement "Early and undesired pregnancies created several barriers to secondary school leading to early school dropout of girls," refer only to girls in the sample or female students more generally?
- The quote used in this section is not directly related to sexual or reproductive health outcomes. A quote from a female adolescent (either a current student or an out-of school student) about pregnancy would be a better fit for this section.

Pg 14: There is a typo: "non-existing" should be changed to "non-existent".

Pg 15: "...labor market skills" to "...skills relevant to the labor market".

Pg 16:
- "...hypothetical school fees" to "...money that could have been allocated to school fees".
- When referring to "the family business", is it the case that every family in the sample had their own enterprise?

Pg 17: More justification is needed for how “Motivational constraints” is different than “Opportunity costs,” given that this section is very clearly describing the opportunity costs of being in school for some students. Personally, I would remove this section and place some of the content into the “Opportunity costs” section.

Pg 18: Change “...held in a shop at the market or outside school to e.g. protect students” to “...held in a shop at the market or elsewhere outside school to protect students”.

1.5 Discussion

General comments: This may help with the background section as well, but there are many quasi-experimental or experimental studies that have attempted to address the barriers to schooling that have not been included as references in this paper. Psaki et al. (2022) “Policies and interventions to remove gender-related barriers to girls’ school participation and learning in low- and middle-income countries: A systematic review of the evidence” (link) has a fairly comprehensive list of these types of studies. Though not all are secondary school-specific, hopefully it will be useful.
