# Supplementary material for: “It’s the poverty”—Stakeholder perspectives on barriers to secondary education in rural Burkina Faso
Source: PLoS One. 2022 Nov 17;17(11):e0277822. doi: 10.1371/journal.pone.0277822 (PMC9671424; doi:10.1371/journal.pone.0277822)
Supplement: Attachment — Submitted filename: Response to Reviewers 28 Jul 2022.docx [file pone.0277822.s005.docx]

**Reviewer comments**

*1.1 Title. The title “Girls were seen as lost investments” is a compelling one, but gives the impression that the paper as a whole will be about barriers to secondary schooling for (primarily) girls. The authors may want to consider using a different quote for their title.*

We agree with the Reviewer and have now changed the title as recommended. We believe the new quote in the title better reflects our findings and the paper as a whole:

“It’s the poverty” - stakeholder perspectives on barriers to secondary education in rural Burkina Faso” (p.1, revised manuscript)

*1.2 Introduction. Pg 4: Describe upfront who counts as a stakeholder in this context.*

We have now clarified the term in the Introduction, as recommended by the Reviewer:

“We defined stakeholders broadly as those who “can effect or [are] affected by the achievement of the organization’s objectives” [23]. We included students, dropout students, parents of enrolled and dropout students, teachers, as well as other relevant stakeholders. In contrast to prior studies in developing settings [24, 25], we included adolescents and youth who are out-of-school and their parents. In doing so, we included the perceptions of some of the most disadvantaged households [26].” (p. 4, revised manuscript)

Reference:

Freeman RE, Reed DL (1983). Stockholders and stakeholders: A new perspective on

corporate governance. *California Management Review, 25*(3), 91.

*1.3 Materials and Methods. Pg 5: Under “Study Area”, for the statements about the predominant occupations of residents, ethnic groups, and languages in the Nouna HDSS, are there any citations that you can provide?*

We thank the Reviewer for this suggestion and have now provided additional citations. In particular, the article by Sié et al. (2010) provides a detailed HDSS profile. We have added this reference to the revised text, which now says:

“While the official language of instruction in the study area is French, the Dioula language serves as a ‘lingua franca’, permitting communication between the different ethnic groups [27]. Additional details on the general population living in the HDSS area are provided elsewhere [16, 27].” (p. 5, revised manuscript)

References:

[16] Werner LK, Jabbarian J, Kagoné M, McMahon S, Lemp J, Souares A, et al. "Because at school, you can become somebody" - The perceived health and economic returns on secondary schooling in rural Burkina Faso. *PLOS ONE*. 2019;14(12):e0226911.

[27] Sié A, Louis V, Gbangou A, Müller O, Niamba L, Stieglbauer G, et al. The Health and Demographic Surveillance System (HDSS) in Nouna, Burkina Faso, 1993-2007. *Glob Health Action*. 2010;3. doi: 10.3402/gha.v3i0.5284.

*Pg 6: Though the authors address in the “Study Limitations” section that there may be bias in their data due to the small sample size, more can be done to address possible sources of bias. For example, providing more detail about the total number of participants invited to interview by participant type (in-school adolescent, out-of-school adolescent, parent, teacher, etc. by gender and other sociodemographic characteristics, if available) and the total number of rejections broken down by those same characteristics would be helpful to understand where bias may lie.*

Thank you for this suggestion. We drew the interviewees from five randomly selected secondary schools out of 28 possible secondary schools within the 58 villages in the Nouna HDSS area. Per chosen school 8 interviewees were sampled. They were invited to participate by members of HDSS or school staff. Among those, one selected participant could not be interviewed due to migration of the HDSS area. We have now clarified this in the Methods section of the paper:

“Respondents were randomly selected by data collectors from the most recent class lists available with the support of school staff as needed (e.g., to reach adolescents and youth who dropped out of school). One dropout could not be interviewed due to migration out of the HDSS and two transcripts were incomplete.” (p.7, revised manuscript)

*The sentence in Page 7 under “Study sample” that begins with “Respondents were randomly selected...” would be better placed under the “Study Design” subsection.*

We have now moved the sentence as recommended by the Reviewer.

*More detail needs to be provided about the second stage sampling – e.g. were there possible sources of bias in the selection of the second stage respondents, given that local study team members were the ones who guided the identification of possible participants?*

We have now further clarified the second stage sampling, where we write:

“… the identification of potential study participants in the second stage of the study was guided by local study team members, which may introduce sampling bias into our results. Data collectors may prefer participants who are, for instance, more similar in terms of background characteristics and may be more likely to be available to them. Nevertheless, study participants represented a wide range of demographic characteristics, including with regard to mother tongue and socio-economic status.” (p. 25, revised manuscript)

*Describe the pilot study first, prior to any description about the first stage sampling.*

We have moved the corresponding sentence as recommended by the Reviewer.

“After a first round of pilot interviews, we improved our study instruments for comprehensibility and, based on preliminary results, further fine-tuned the questionnaires used in the study.” (p. 6, revised manuscript)

*Pg 7: – Under “Interviews”, were the local experts hired and managed directly by the
study team, or were they associated with a survey firm of some kind?*

Local experts were hired and managed directly by the study team as opposed to e.g., a survey firm. We have now further clarified this in the paper, where we write:

“Experienced local interviewers, who were hired and managed directly by the CRSN,

were trained in Nouna, Burkina Faso, for two days.” (p. 6-7, revised manuscript)

*There is a redundant citation under “Study Sample” ([30]).*

Thank you for catching this. We have now removed the redundant citation.

*Pg 8: Provide a formal definition of “resilience” and define what “maladaptive coping strategies” are in this context.*

We have added definitions for resilience and coping strategies in the Methods section:

“… the model considers ‘stressors’ which challenge an individual’s resilience defined as the capability to maintain or regain mental stability [35, 36]. Coping of a person can be described as “conscious volitional efforts to regulate emotion, cognition, behavior, physiology, and the environment in response to stressful events or circumstances” [37]. These mechanisms have been categorized in the literature as e.g., adaptive vs. maladaptive strategies. Maladaptive coping strategies have been specified as harming [38]. In our application, we hypothesize that circumstances and individual resilience influence what a barrier to secondary school *means* to households and adolescents, rather than deterministic quantitative criteria.” (p. 8, revised manuscript)

“We also looked for behaviors we thought could be indicative of maladaptive coping strategies and may cause short term stress relieve but have detrimental effects to the individual in the future. Maladaptive coping strategies may include, for instance, substance abuse, risky (sexual) behavior, and social withdrawal.” (p. 9, revised manuscript)

*1.4 Result. General comments: First, it’s unclear where many of the accounts from - students, out of school adolescents, parents, teachers, or others. This is important for some of the views on barriers to education (e.g. the accounts of students being envious of out-of-school peers in
the labor force). If the descriptions came from multiple types of stakeholders, it may benefit
the reader to be more explicit.*

Apologies for the lack of clarity. We have now clarified the type of stakeholder throughout the Results section – e.g.:

“Teachers reported school absenteeism due to several health concerns, in particular with regards to sexual and reproductive health.” (p. 13, revised manuscript)

“Female students also wished for single sex toilets to stop the “teasing” at toilets between the genders; with the exception of madrassas (Islamic schools), which would have guaranteed separate toilets for women and men.” (p. 14, revised manuscript)

“Nightfall and battery life were reported by students and teachers as limiting factors to learning since electricity, torches, or solar panels were not widely available at home.” (p. 18, revised manuscript)

*Second, a clearer distinction should be made about whether some barriers primarily affect
boys or affect boys and girls more-or-less equally. For example, the account about the salience of the benefits of not being a student by observing the relative wealth of out-of-school peers seems like a barrier that affects mostly boys, since girls are expected to be homemakers according to the interviews. The language used when describing this observation is gender-neutral, even though the barrier is not.*

We thank the Reviewer for this suggestion and have now clarified this in the paper – e.g.:

“Young men mentioned, for instance, the appeal of planting sesame seeds, working in mines, or migrating to Ivory Coast to engage in cacao farming.” (p. 13, revised manuscript)

“Early and undesired pregnancies created several barriers to secondary school leading to early school dropout of girls. Two interviewed young women reported to have quit school because of pregnancy. Teachers reported that young women who were pregnant faced teasing and contemptuous language or behavior directed at their pregnancy.” (p. 13, revised manuscript)

*Lastly, there is an excessive use of the word “would” in much of this section. Many of the
accounts are of events or observations that have already happened, but this passive language makes it sound like some of these events have not happened yet. The language needs to be amended.*

We have now reduced the use of the word “would” in this section – e.g.:

“Households and adolescents saw few career opportunities other than working in agriculture.” (p.13, revised document)

“Young women were encouraged to specialize in domestic skills (homemaking) during early adolescence, which often implied becoming a stay-at-home mom instead of having a career.” (p.16, revised document)

“According to one school headmaster, investments in daughters’ schooling paid little dividends in the minds of parents because young women were married to be “housewives” and ultimately drop out of school anyway …” (p.16, revised document)

*Pg 12: There is a typo: “–;” should be changed to “–”.*

We have now corrected the typo in the paper.

*Pg 13: – Does the statement “Early and undesired pregnancies created several barriers to secondary school leading to early school dropout of girls,” refer only to girls in the sample or female students more generally?*

We have now clarified this point in the paper. We obtained confirmation of two young women in our sample to have quit school as a direct result of becoming pregnant:

“Early and undesired pregnancies created several barriers to secondary school leading to early school dropout of girls. Two interviewed young women reported to have quit school because of pregnancy.“ (p. 14, revised manuscript)

*The quote used in this section is not directly related to sexual or reproductive health outcomes. A quote from a female adolescent (either a current student or an out-of school student) about pregnancy would be a better fit for this section.*

We thank the Reviewer for this excellent suggestion. We have now used an alternative quote which more directly illustrates the gist of this sub-section in the paper:

“As I got pregnant, I said I would drop out this year.” *–* Dropout, female, 17 years

*Pg 14: There is a typo: “non-existing” should be changed to “non-existent”.*

We thank the Reviewer for bringing this to our attention. This word has been corrected.

*Pg 15: “...labor market skills” to “...**skills relevant to the labor market”.*

The sentence has changed as suggested.

*Pg 16: “...hypothetical school fees” to* *“...money that could have been allocated to school
fees”.*

The sentence has now been edited accordingly.

*When referring to “the family business”, is it the case that every family in the sample had their own enterprise?*

We thank the Reviewer for this comment. The reported kind of work included subsistence farming, or work in small shops or small market stalls e.g. to sell food. Whether this in every case was owned by the family or family members were employed is not clearly distinguishable in every case. We have now clarified this in the paper, where we write:

“Parents reported not sending their children to school based on subjective benefit-cost assessments of attending school and instead instructed them to work at home, in small shops or small market stalls, or engage in other paid work. Parents invested money that could have been allocated to school fees in economic or domestic activities (e.g., purchasing animals for farming or breeding).” (p. 17, revised manuscript)

*Pg 17: More justification is needed for how “Motivational constraints” is different than “Op-
portunity costs,” given that this section is very clearly describing the opportunity costs of being in school for some students. Personally, I would remove this section and place some of the content into the “Opportunity costs” section.*

We agree with the Reviewer and have moved the text to the section on opportunity costs.

*Pg 18: Change “...held in a shop at the market or outside school to e.g. protect students” to*
*“...held in a shop at the market or elsewhere outside school to protect students”.*

The sentence has now been edited accordingly.

*1.5 Discussion. General comments: This may help with the background section as well, but there are many quasi-experimental or experimental studies that have attempted to address the barriers to schooling that have not been included as references in this paper. Psaki et al. (2022) “Policies and interventions to remove gender-related barriers to girls’ school participation and learning in low- and middle-income countries: A systematic review of the evidence” (link) has a fairly comprehensive list of these types of studies. Though not all are secondary school-specific, hopefully it will be useful.*

We thank the Reviewer for pointing us to this helpful reference. We now cite it and discuss several papers from this review in our Discussion section of the paper – e.g.:

“Rheinländer et al., for instance, highlighted the role of sanitation for girls’ secondary schooling rates [55]. A recent systematic review identified several promising interventions to remove barriers to girls' school participation and learning in low-resource settings, including lack of water and sanitation [56].” (p. 23, revised manuscript)

“… there is a need for well-designed studies on tackling barriers to schooling in settings where secondary schooling is persistently low [56, 66].” (p. 24, revised manuscript)

“In terms of nutritional environment, school-based food assistance could provide students to have at least one proper meal a day, further improving both health and education outcomes jointly [57-59].” (p. 24, revised manuscript)

References:

Aurino E, Gelli A, Adamba C, Osei‐Akoto I, Alderman H (2020). Food for thought? Experimental evidence on the learning impacts of a large‐scale school feeding program in Ghana. *Journal of Human Resources*. doi: 10.3368/jhr.58.3.1019-10515R1.

Kazianga H, de Walque D, Alderman H (2009). Educational and health impacts of two school feeding schemes: Evidence from a randomized trial in rural Burkina Faso. World Bank, 2009. doi: 10.1596/1813-9450-4976.

Psaki S, Haberland N, Mensch B, Woyczynski L, Chuang E (2022). Policies and interventions to remove gender‐related barriers to girls' school participation and learning in low‐ and middle‐income countries: A systematic review of the evidence. *Campbell Systematic Reviews*; 18(1). doi: 10.1002/cl2.1207
