# Supplementary material for: “It’s the poverty”—Stakeholder perspectives on barriers to secondary education in rural Burkina Faso
Source: PLoS One. 2022 Nov 17;17(11):e0277822. doi: 10.1371/journal.pone.0277822 (PMC9671424; doi:10.1371/journal.pone.0277822)
Supplement: Attachment — Submitted filename: Response to Reviewers 15 Sep 2022.docx [file pone.0277822.s006.docx]

**Reviewer comments**

*Reviewer #1: Thank you all for addressing my comments. I think this is a nice improvement on the previous version of the paper and you should be commended for your hard work.*

We thank the Reviewer for these comments.

*Reviewer #2: Girls were seen as lost investments” – stakeholder perspectives on barriers to secondary education in rural Burkina Faso.*

The Reviewer writes “*Girls were seen as lost investments…”*, which refers to a previous quote in the title of the paper. The quote in the title has been replaced since then, however, to address comments included in the first decision letter we received from the journal (June 2022). This suggests that the Reviewer may, unfortunately, have received an older version of the paper as opposed to our first revision. As a result, not all comments from Reviewer #2 may be entirely applicable. Nevertheless, the comments from Reviewer #2 are still very helpful and we have now incorporated these in a second “harmonized” revision of the paper.

“It’s the poverty” - stakeholder perspectives on barriers to secondary education in rural Burkina Faso” (p.1, title, revised manuscript)

*Thank you for giving me the opportunity to review this article. It is an interesting article and relevant for not only the context of Burkina Faso but also for similar countries. The paper is conceptually sound and well-written, but I think it needs some minor revisions before it can be published. The following are my suggestions.*

We thank the Reviewer for these comments.

*“These large existing gaps and inequalities in secondary school completion are likely to be further exacerbated by the COVID-19 pandemic, which has resulted in dramatic losses in the income and livelihoods of poor households [19] and temporary school closures in many settings [20].”. This point is relevant, but COVID 19 impact seems to be relatively less a major issue in most African countries, including Burkina Faso. I would suggest also adding and discussing an aspect which is more relevant to education, that is insecurity in many areas of the country. Currently, security issues seem to be more relevant to education than COVID 19 is. How does that affect girls’ education?*

Thank you for this suggestion. At the time of data collection in April 2018, the security situation in the study area was just beginning to worsen. Since then, a coup d'état was launched in Burkina Faso in January 2022 and violence around our study area has picked up substantially in the past few years (Nouna area, Boucle du Mouhoun region). Nevertheless, we agree with the Reviewer that this aspect has become more relevant to education (Bene 2022). We have therefore further discussed the security situation in the country, e.g.:

“These large existing gaps and inequalities in secondary school completion are likely to be further exacerbated by the COVID-19 pandemic and worsening security situation, which have resulted in losses of income and temporary school closures in many settings, including in Burkina Faso [20, 21].” (p. 3, revised manuscript)

“Sixth, since the time of data collection, a coup d'état was launched in Burkina Faso (January 2022) and the security situation has worsened in the study area. Our results, particularly around security as a potential barrier to schooling, may not be generalizable to different periods.” (p. 25, revised manuscript)

References:

Bene K. Gauging secondary school students' terrorism‐related resilience in the Sahel region of Burkina Faso: a quantitative study. *Psychology in the Schools*. 2022. doi: 10.1002/pits.22779.

*After reading the introduction, I could not find out the rationale for the study. In relation to the topic, what is literature saying and what is not yet known? It is important to bring out this point because it highlights the need for your study.*

We have now further clarified the rationale in the Introduction section of the paper, as recommended by the Reviewer. First, despite a few recent studies from Burkina Faso, relatively little remains known about the most effective strategies to remove barriers to secondary schooling, particularly in settings where schooling is very low (Psaki S et al., 2022). Second, most prior studies from lower-income countries exclude out-of-school adolescents (such as the Global School-based Student Health Survey). We include the perceptions of a wide range of stakeholders, including out-of-school adolescents.

“Relatively little remains known, however, regarding barriers to secondary schooling, particularly in contexts where schooling is very low. In a recent review of interventions to remove barriers to girls’ schooling in low- and middle-income countries, for example, only two out of >80 studies were conducted in Burkina Faso [22]. Both studies assessed broad efforts to improve the school environment, such as constructing entire schools and additional classrooms, and were focused on the primary school level [23, 24].” (p.3, revised manuscript)

“In contrast to prior studies from low- and middle-income countries [25, 26], we included adolescents and youth who are out-of-school and their parents. In doing so, we included the perceptions of some of the most disadvantaged households [27].” (p.4, revised manuscript)

References:

Psaki S, Haberland N, Mensch B, Woyczynski L, Chuang E. Policies and interventions to remove gender‐related barriers to girls' school participation and learning in low‐ and middle‐income countries: A systematic review of the evidence. *Campbell Systematic Reviews*. 2022;18(1). doi: 10.1002/cl2.1207.

*In presenting the educational context of the country, I suggest to link it to your topic. For example, are there policies for girls’ education at the level of post primary education? How does the government promote girls’ education at this level?*

Thank you for this suggestion. We have now provided additional educational context of the region, as recommended by the Reviewer, including in the Introduction and Methods sections. Specifically, we have further highlighted existing evidence on barriers to schooling (UNICEF 2017) and on existing strategies to remove barriers to schooling (Psaki S et al., 2022).

“Relatively little remains known, however, regarding barriers to secondary schooling, particularly in contexts where schooling is very low. In a recent review of interventions to remove barriers to girls’ schooling in low- and middle-income countries, for example, only two out of >80 studies were conducted in Burkina Faso [22]. Both studies assessed broad efforts to improve the school environment, such as constructing entire schools and additional classrooms, and were focused on the primary school level [23, 24].” (p.3, revised manuscript)

“At the national level, commonly reported reasons for school absenteeism include a lack of financial means and a lack of interest in attending school (school is “not deemed necessary”) [34]. These findings suggest that poverty and the perceived benefits of going to secondary school may play an important role in decision-making around school participation and performance [35-37].” (p. 6, revised manuscript)

References:

Psaki S, Haberland N, Mensch B, Woyczynski L, Chuang E. Policies and interventions to remove gender‐related barriers to girls' school participation and learning in low‐ and middle‐income countries: A systematic review of the evidence. *Campbell Systematic Reviews*. 2022;18(1). doi: 10.1002/cl2.1207.

UNICEF. Rapport d’état du système éducatif national du Burkina Faso, Pour une politique nouvelle dans le cadre de la réforme du continuum d’éducation de base. Pôle de Dakar de IIPE: UNESCO; 2017 [available at: <https://unesdoc.unesco.org/ark:/48223/pf0000253643>]

*The study design is clear and strong. However, I suggest you make it clear that your questionnaire is designed based on literature (if that is so), and you can provide a few references accordingly. The questionnaire can even be attached to the appendix if there are no constraints in relation to wordcount.*

All questionnaires were developed by the study team. The questionnaires covered hypothesized barriers to secondary schooling in the region and were based on readings of the published and grey literature as well as prior research involving out-of-school adolescents in the study area (*Trop Med Int Health* 2020, *Glob Health Action* 2010). We have now clarified these points in the Methods section of the paper. Additionally, as recommended by the Reviewer, we have now added all questionnaires to the Appendix (**S1** **File**).

“We developed questionnaires for our qualitative semi-structured interviews separately for each category of stakeholders (e.g., enrolled students, out-of-school adolescents, parents, and teachers). The questionnaires covered hypothesized barriers to secondary schooling, using *a priori* developed categories of barriers (described below), and were based on readings of the literature and prior research in the study area [28-30]. All questionnaires are available in S1 File. (p. 6, revised manuscript)

“S1 File. Study questionnaires”

References:

De Neve JW, Karlsson O, Canavan CR, Chukwu A, Adu-Afarwuah S, Bukenya J, Darling AM, Harling G, Moshabela M, Killewo J, Fink G, Fawzi WW, Berhane Y. Are out-of-school adolescents at higher risk of adverse health outcomes? Evidence from 9 diverse settings in sub-Saharan Africa. *Trop Med Int Health*. 2020 Jan;25(1):70-80.

Sié A, Louis V, Gbangou A, Müller O, Niamba L, Stieglbauer G, et al. The Health and Demographic Surveillance System (HDSS) in Nouna, Burkina Faso, 1993-2007. *Glob Health Action*. 2010;3. doi: 10.3402/gha.v3i0.5284.

*Reviewer #3: This is an interesting paper which offers some important insights into the day-to-day barriers to education in rural Burkina Faso. I am reviewing the paper for the first time but I can see that the authors have made good efforts to respond to earlier review comments and have provided a comprehensive overview of how they have responded to these points. I do not feel that any of the findings are surprising or particularly new - but nonetheless they add to a body of literature that captures the complexity of reasons why young people may discontinue attending school in rural communities.*

We thank the Reviewer for these comments.

*A number of relatively small points I think need addressing: The use of 'dropout'; 'dropouts' as labels is derogatory and in my opinion should be avoided.*

Thank you for pointing this out. We have now removed the term throughout the entire paper and have instead used more neutral terms (such as “out-of-school” adolescents). There were two exceptions in the text, however, where we have kept the term drop-out. Specifically, because the term was used as a search term in our literature search (p. 8) and because a respondent’s quote translated to “As I got pregnant, I said I would drop out this year.” (p. 15).

“We included students, out-of-school adolescents, parents of enrolled and out-of-school adolescents.” (p.4, revised manuscript)

“From each selected school, a total of eight interviewees were selected: two enrolled students, two out-of-school adolescents (who did not attend school between one month and one year prior to the interview), one parent of an enrolled student, one parent of an out-of-school adolescent, as well as two teachers.” (p. 6, revised manuscript)

“Eight out of nine out-of-school adolescents reported being “currently employed” at the time of the study.” (p. 11, revised manuscript)

*There is some conflation/confusion throughout the paper between what is presented as a 'qualitative study' and issues of sampling and representation. At one stage participants are described as being ' purposefully randomly selected'; purposive and random sampling are entirely different processes so there needs to be some greater clarity here.*

Thank you for bringing this to our attention. We agree with the Reviewer and have now clarified the corresponding sentence, where we write:

“First, we randomly selected five out of 28 secondary schools in the Nouna HDSS.” (p. 26, revised manuscript)

*While the main the research method was semi-structured interviews - you mention a 'group adapted questionnaire (p.6) - which is described as distinct from the demographic questionnaire. It was unclear to me what this was and what it added to the data*

We apologize for the confusion. We developed questionnaires for our qualitative semi-structured interviews separately for each category of stakeholders (e.g., enrolled students, out-of-school adolescents, their parents, and teachers). These were our study instruments in this qualitative study. An overview of results from the qualitative interviews are presented in **Table 2** of the paper. The questionnaires have also been added to the Appendix **(S1 File**).

Additionally, to assess the background characteristics of respondents who participated in our qualitative interviews (above), we collected basic quantitative socio-demographic characteristics such as age, sex, parental education, primary occupation of the household, and language. An overview of these socio-demographic characteristics is shown in **Table 1** of the paper. We have now further clarified these points, where we write – e.g.:

“We developed questionnaires for our qualitative semi-structured interviews separately for each category of stakeholders (e.g., enrolled students, out-of-school adolescents, parents, and teachers). The questionnaires covered hypothesized barriers to secondary schooling, using *a priori* developed categories of barriers (described below), and were based on readings of the literature and prior research in the study area [28-30]. All questionnaires are available in S1 File. In addition, we collected basic quantitative socio-demographic characteristics from all participants (such as age and parental education).” (p. 6, revised manuscript)

“S1 File. Study questionnaires”

*In terms of ethics and consent - while there is talk of asking adults for their consent and for the consent for participation for young people below the age of 18, there is no mention of young people giving consent to participate in the study or any indication of whether or not they were given any choice about their participation.*

Written informed consent was obtained from every interviewee or parent for interviewees younger than 18 years. For minors, we sought adult consent in advance of the child’s assent. We defined assent as involving the child to the extent compatible to his or her maturity and with local cultural norms (Cheah & Parker 2014). Although no child in our study expressed reservations about participating in the study, this does not imply that the child necessarily had the final say in the decision to enroll in the study. Enrolment was based on the best interests of the child (World Health Organization 2018). The study was pre-registered and approved by the Institutional Ethics Committee of the Nouna Health Research Center (CRSN), in Burkina Faso, and the Heidelberg University Hospital Ethics Committee, in Germany.

“Written informed consent was obtained from every interviewee or parent for interviewees younger than 18 years. Adult consent was obtained in advance of the child’s assent for minors”. (p. 7, revised manuscript)

References:

Cheah PY, Parker M. Consent and assent in paediatric research in low-income settings. *BMC Medical Ethics*. 2014;15(1). doi: 10.1186/1472-6939-15-22.

World Health Organization. Guidance on ethical considerations in planning and reviewing research studies on sexual and reproductive health in adolescents. 2018. [Available at; <https://apps.who.int/iris/handle/10665/273792>].

*The representation of participants via quotes is unclear - so just having the gender and age of a participant does not adequately distinguish between them nor does it tell the reader whether we are hearing from the same young person or whether they are different voices.*

Thank you for this suggestion. In our paper, we use illustrative quotes from a wide range of different voices, representing all categories of stakeholders in the study, including in- and out-of-school adolescents, their parents, teachers, school headmasters, and other respondents. Following each quote, we then provide the sex, age, and category of each respondent. For instance, “Out-of-school, female, ages 17 years”. This approach allows the reader to differentiate between the different categories of stakeholders and key demographic characteristics. We also present supplementary quotes in the Appendix (**S1 Text**).

Nevertheless, to address the Reviewer’s comment, we have now added the first letter of a pseudonym after each quote to further differentiate between the different voices *within* each category of stakeholders (e.g., “I” for Isabelle). These pseudonyms allow the reader to track the comments of participants across quotes. For instance, to know whether we are hearing from the same – or a different – out-of-school young woman ages 17 years. We decided against using full names as pseudonyms since full names can have specific social class and ethnic connotations (Wiles 2013). We have now clarified this in the Methods section and added the first letter of pseudonyms after each quote in the Results section - e.g.:

“When showing quotes in the text, we added the first letter of a pseudonym after each quote to protect the privacy of interviewees and allow the reader to track the comments of interviewees across quotes.” (p. 7, revised manuscript)

“*It’s the poverty*.” – I, out-of-school, female, 17 years

References:

Wiles, Rose. “Thinking ethically: approaches to research ethics.” What are Qualitative Research Ethics? London: Bloomsbury Academic, 2013. 9–24.
